# Investigation into Clearance of Organic Compounds from Biomanufacturing Process Streams during Ultrafiltration/Diafiltration

**DOI:** 10.1007/s11095-026-04050-2

**Published:** 2026-03-24

**Authors:** Noemí Dorival-García, Anna Mulligan, Ronan Hayes, Charles Felice, Aidan Sexton, Ping-Ping Wang, Jonathan Bones

**Affiliations:** 1 Characterisation and Comparability Laboratory, The National Institute for Bioprocessing Research and Training (NIBRT), Foster Avenue, Mount Merrion, A94 X099 Co. Dublin Ireland; 2 Manufacturing Science and Technology, Johnson & Johnson Innovative Medicine, Barnahely, P43 FA46 Co. Cork Ireland; 3 Material Sciences at Discovery, Product Development & Supply, Johnson & Johnson Innovative Medicine, 200 Great Valley Parkway, Malvern, PA 19355 USA; 4 Material Sciences at Biotherapeutics Development & Supply, Johnson & Johnson Innovative Medicine, Barnahely, P43 FA46 Co. Cork Ireland; 5 School of Chemical and Bioprocess Engineering, University College Dublin, Dublin 4, Belfield, D04 V1W8 Ireland

**Keywords:** Liquid chromatography-mass spectrometry, Process equipment related leachables, Regression models, Ultrafiltration/diafiltration, Vortex-assisted liquid–liquid microextraction

## Abstract

**Objective:**

Ultrafiltration and diafiltration (UF/DF) operations have been demonstrated to clear leachables from drug substance, however there is limited data available. Consequently, comprehensive and systematic characterization of leachables clearance during UF/DF is required and essential.

**Methods:**

To achieve this, the reduction capacity for 28 selected organic compounds spiked into 3 different proteins during UF/DF processes was investigated using liquid chromatography high-resolution mass spectrometry. Selection of compounds was based on their presence in representative biomanufacturing processes.

**Results:**

Most compounds (24) showed clearance over 98% across the process for the 3 protein materials. The specific protein characteristics and process parameters for each protein had a minimal impact on clearance, with sieving coefficients essentially the same for each one of the 3 protein processes. The sieving coefficient is a parameter that characterizes clearance of compounds during UF/DF. Physicochemical properties of the compounds under study significantly influenced their clearance, with the octanol–water coefficient (Log P) being the most crucial factor. Compounds with Log P < 4 had sieving coefficients close to ideal clearance, and compounds with Log P > 7 showed lower but still significant clearance (> 93%). Other important parameters were established to be molecular weight, polarizability and solvent accessible surface area. Modelling tools based on Orthogonal Partial Least Squares (OPLS) regression were created to predict sieving coefficients.

**Conclusions:**

The present work has created a strong background to describe the ability of UF/DF to remove potential organic leachables. Application of these modelling approaches becomes critical to support product safety assessments. Demonstration of significant removal along UF/DF operations confirms risk reduction of leachables coming mostly from upstream stages.

**Graphical Abstract:**

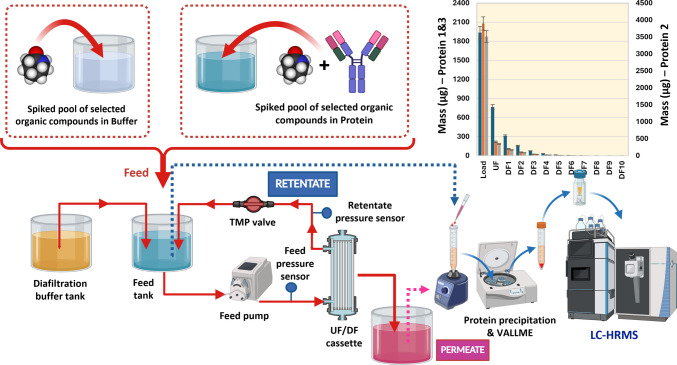

**Supplementary Information:**

The online version contains supplementary material available at 10.1007/s11095-026-04050-2.

## Introduction

Implementation of SUTs in biopharmaceutical manufacturing processes has brought advantages to the industry [1]. The evolution of SUTs began in the early 2000 s when the biopharmaceutical industry recognized the need for more flexible, cost-effective and rapid manufacturing solutions. Prevalence of SUTs in the biopharmaceutical industry has increased significantly over the past two decades. They have transformed conventional manufacturing of therapies such as mAbs and facilitated production of emerging modalities like antibody–drug conjugates, vaccines, and cell and gene therapy products [2].

Despite refinement of SUTs driven by advancements in materials science, manufacturing processes and construction materials, challenges concerning PERLs remain [2]. PERLs often originate from polymers, elastomers and other materials, where they may arise from additives (e.g., antioxidants, slip agents, plasticizers, antistatic agents, UV and heat stabilizers, colorants and lubricants) and processing aids [3]. PERLs are leachables, which according to the FDA, are compounds that leach into the biomanufacturing solutions from SUTs under working conditions, unlike extractables, which can be extracted from SUTs in the presence of a solvent under extreme conditions, mainly time and temperature.

Consequently, PERLs can migrate from SUTs into the bioprocessing solutions and potentially reach the final product [4–6], representing a risk to patient safety and regulatory compliance during commercial-scale operations [7] as PERLs can also negatively impact process performance, especially during upstream cell culture operations [8, 9]. Consequently, the presence of PERLs requires screening and thorough assessment [10]. Aside from potential safety concerns, PERLs have received widespread attention from a product quality and stability standpoint. Unwanted species can form from unreacted polymerization components, directly from the additives used in construction, from catalysts used during production, or from material degradation during storage, transport, or gamma irradiation [11].

To standardize and align best practice for extractables studies, BioPhorum [12] and US Pharmacopeia (USP < 665 > [13] and < 1665 > [14]), have developed and published documents with a view to standardizing protocols for potential leachables testing of SUTs. These guidelines directly address many of the noted issues and are expected to greatly improve the uptake, availability and quality of data for quality risk management practices.

While thorough characterization of E&Ls is extremely beneficial for creating databases and increasing awareness of their possible occurrence in the final product, conducting routine tests for every substance in each manufacturing lot is impractical and not realistic [15]. Suitable implementation of safety risk assessments should be based on an assisted strategy that considers, for instance, the impact of the downstream clearance potential to avoid any superfluous testing. Downstream processing purifies the product by removing cells, cell debris and other components, and PERLs are also removed. Availability of sufficient, evidence-based information on PERL clearance becomes critical in evaluating the scope of current PERL testing strategies. For example, PERLs which exhibit effective removal during downstream purification may be assessed as low risk.

UF/DF is the final downstream manufacturing step for most biologic drug substance processes and is the industry standard for concentration and buffer exchange of protein and peptide-based therapeutics [16]. Substances that cannot be removed during UF/DF pose a greater safety risk due to the increased likelihood of their persistence in the final drug product. The potential for these impurities to interact with the therapeutic molecule also raises the level of concern [17, 18].

Several studies have demonstrated significant clearance of organic impurities from biomanufacturing processes using UF/DF [19–21], achieving reduction capacity of up to 1000-fold, and showing that compound clearance depends on their physicochemical properties, *e.g.,* Log P.

Nevertheless, it remains essential to create a platform that can scientifically predict process clearance by considering various factors, like dependence on the physicochemical characteristics of the protein product and PERLs, along with the parameters of the UF/DF process. Such a model would be a useful tool in future risk assessments.

Here, the determination of the reduction capacity of UF/DF operations for removing organic PERLs from process streams was systematically investigated. A total of 28 organic compounds were selected, comprising additives and degradation products commonly found as PERLs from plastic materials, whose relevance is linked to their potential impact on drug product safety. To mention some significant examples, nitrosamines are known genotoxic impurities from the manufacture of angiotensin II receptor blocker drugs [22]. Bisphenol A is widely used as a raw material to produce polycarbonate plastics, epoxy resins, and lacquer coatings [23], but is an estrogenic chemical, which can modify natural endocrine functions by binding to the estrogen receptor, leading to adverse effects on human health, such as the development of breast cancer, endometriosis and infertility. Another selected cytotoxic PERL was bDtBPP, a breakdown product of the antioxidant additive Irgafos® 168 used widely for polymeric films in SUTs, being a remarkably potent inhibitor of cell growth for sensitive cell lines [24].

Analytical methods were developed using LC-HRMS to track the behaviour of the selected organic compounds during UF/DF processes for three different mAb formulations. Compounds were measured in highly concentrated protein solutions (up to 180 g L^–1^), which represents an analytical challenge since the presence of protein can cause significant interference to the measurement of small molecules. Sample preparation using a novel VALLME method was optimized to minimize matrix effects and enhance analytical performance.

Furthermore, multivariate statistical techniques were employed to create mathematical models that describe and forecast the clearance behavior of the compounds throughout UF/DF procedures, relying on their physicochemical properties. An OPLS model was developed to predict sieving coefficients with 98.6% prediction power. These models may represent an impactful contribution to the biopharmaceutical industry by supporting potential modification of PERL testing strategies, using predictive modelling to avoid the need to generate process-specific clearance data, reducing the need for assay development and qualification. Application of PERL clearance modelling may also support more targeted process development to enhance removal capacity, facilitating management of safety and product stability risk assessments by process design.

## Materials and methods

### Chemicals and Materials

Table I shows the list of 28 organic compounds selected for this study, including internal standards: 2-(2-hydroxy-5-methylphenyl)benzotriazole (Tinuvin P), chosen according to the ASTM D-6042 [25] for compounds determined under positive polarity, and Bisphenol A-d16 for compounds under negative polarity. Pierce FlexMix solution for MS calibration was purchased from Thermo Fisher Scientific (Bremen, Germany). Deionized water (18.2 MΩ) was produced by a Sartorius Stedim Biotech Arium 61316 system (Göttigen, Germany). SoloVPE fibrettes and plastic vessels were acquired from Repligen (Waterford, Ireland) to measure protein concentration using the CTech SoloVPE System. Solvents and chemicals for extractions and UF/DF processes, as well as mobile phases and additives for UHPLC–MS analysis, were purchased from Fisher Scientific (Dublin, Ireland). Three mAbs (P1, P2, and P3) were supplied by Johnson & Johnson Innovative Medicine, obtained from protein A affinity chromatography eluate, post UF/DF, and VIN processing stages, respectively (at $\ge 90\%$ purity).

**Table I:** Organic leachables: Properties, applications, parent ion and polarity used for MS quantification

#	CAS	Name	Abbreviation	Formula	Supplier	Application	MW^(*)^	Log P^(*)^	Exact mass	Polarity	Parent ion	
											[M + H]^+1^	[M—H]^−1^
Training set												
1	62–75-9	N-Nitrosodimethylamine	NDMA	C_2_H_6_N_2_O	Merck	Impurity from drugs manufacturing	74.08	0.04	74.0480	Positive	75.0558	
2	872–50-4	N-Methylpyrrolidone	NMP	C_5_H_9_NO	Merck	Solvent, adhesive and sealant	99.13	−0.36	99.0684	Positive	100.0762	
3	105–60-2	ε-Caprolactam	CAP	C_6_H_11_NO	Merck	Monomer from polyamines	113.16	0.31	113.0841	Positive	114.0919	
4	100–75-4	1-Nitrosopiperidine	NPIP	C_5_H_10_N_2_O	Merck	Impurity from drugs manufacturing	114.15	0.89	114.0793	Positive	115.0871	
5	104–90-5	5-Ethyl-2-methylpyridine	EMP	C_8_H_11_N	Merck	Copolymer	121.18	1.85	121.0891	Positive	122.0970	
6	149–30-4	2-Mercaptobenzothiazole	MBZ	C_7_H_5_NS_2_	Merck	Vulcanization accelerator (rubber)	167.24	2.88	166.9863	Negative		165.9785
7	103–49-1	Dibenzylamine	DIB	C_14_H_15_N	Merck	Intermediate in polymer synthesis	197.28	3.26	197.1205	Positive	198.1283	
8	80–39-7	N-Ethyltoluene-4-sulfonamide	ETS	C_9_H_13_NO_2_S	Merck	Plasticizer	199.27	1.67	199.0667	Negative		198.0589
9	128–39-2	2,6-Di-tert-butylphenol	6DTBP	C_14_H_22_O	Merck	Antioxidant (degradation product)	206.32	4.76	206.1671	Negative		205.1592
10	3622–84-2	N-Butyl-benzenesulfonamide	BBS	C_10_H_15_NO_2_S	Merck	Plasticizer	213.30	2.13	213.0824	Negative		212.0745
11	128–37-0	2,6-Di-tert-butyl-4-methylphenol	BHT	C_15_H_24_O	Merck	Antioxidant	220.35	5.27	220.1827	Negative		219.1749
12	80–05–7	Bisphenol A	BPA	C_15_H_16_O_2_	Merck	Monomer from polycarbonate	228.29	4.05	228.1150	Negative		227.1072
13	1620–98-0	3,5-Di-tert-butyl-4-hydroxybenzaldehyde	BHT-CHO	C_15_H_22_O_2_	Merck	Antioxidant (degradation product)	234.34	4.47	234.1620	Negative		233.1542
14	1421–49-4	3,5-Di-tert-butyl-4-hydroxybenzoic acid	BHT-COOH	C_15_H_22_O_3_	Merck	Antioxidant (degradation product)	250.33	4.42	250.1569	Negative		249.1491
15	732–26-3	2,4,6-tri-tert-butylphenol	TTB	C_18_H_30_O	Merck	Antioxidant (degradation product)	262.44	6.31	262.2297	Negative		261.2219
16	126–73-8	Tributylphosphate	TBP	C_12_H_27_O_4_P	Merck	Solvent for inactivation of viral lipid envelope	266.32	4.09	266.1647	Positive	267.1725	
17	112–84-5	cis-13-docosenic acid amide (Erucamide)	ERU	C_22_H_43_NO	Merck	Slip agent	337.59	7.76	337.3345	Positive	338.3423	
18	77–90-7	Tributyl O-acetylcitrate	ATBC	C_20_H_34_O_8_	Merck	Adhesive and plasticizer	402.48	3.53	402.2254	Positive	403.2332	
19	7128–64-5	2,5-Bis(5-tert-butyl-benzoxazol-2-yl)thiophene	BBOT	C_26_H_26_N_2_O_2_S	Merck	Optical brightener	430.56	7.58	430.1715	Positive	431.1793	
20	69,284–93-1	Bis(2,4-di-tert-butylphenyl) hydrogen phosphate	bDtBPP	C_28_H_43_O_4_P	Fluorochem	Antioxidant (degradation product)	474.62	9.23	474.2899	Positive	475.2977	
Prediction set												
21	88–99-3	Phthalic acid	PHT	C_8_H_6_O_4_	Merck	Phthalates (plasticizer) degradation product	166.13	1.29	166.0266	Negative		165.0188
22	2432–99-7	11-Aminoundecanoic acid	AUD-COOH	C_11_H_23_NO_2_	Merck	Initiator for type 11 Nylons	201.31	0.23	201.1729	Positive	202.1807	
23	719–22-2	2,6-Di-tert-butyl-1,4-benzoquinone	tBBQ	C_14_H_20_O_2_	Merck	Oxidant and polymerization catalyst	220.31	3.88	220.1463	Positive	221.1542	
24	629–54-9	Hexadecanamide	HEX	C_16_H_33_NO	LGC	Slip agent	255.44	5.45	255.2562	Positive	256.2640	
25	301–02-0	Oleamide	OLE	C_18_H_35_NO	Merck	Slip agent	281.48	6.00	281.2719	Positive	282.2797	
26	57–11-4	Stearic acid	STE	C_18_H_36_O_2_	Merck	Lubricant	284.48	7.15	284.2715	Negative		283.2637
27	5581–32-8	Bisphenol A bis(2,3-dihydroxypropyl) ether	Bis-HPPP	C_21_H_28_O_6_	Merck	Component of epoxy resins	376.44	1.70	376.1886	Positive	377.1964	
28	540–97-6	Dodecamethylcyclohexasiloxane	SIXD6	C_12_H_36_O_6_Si_6_	Merck	Coating agent	444.92	1.15	444.1127	Positive	445.1205	
Internal standards												
29	2440–22-4	Tinuvin P	TINP	C_13_H_11_N_3_O	Merck		225.25	3.19	225.0902	Positive	226.0980	
30	96,210–87-6	Bisphenol A d16	BPA-d16	C_15_D_16_O_2_	Merck		244.39	3.30	244.2155	Negative		241.1935

^(*)^*MW* = molecular weight; *Log P* = octanol/water partition coefficient

### UF/DF Load Sample Preparation

Buffer and protein solutions were spiked with the targeted organic compounds at a final concentration of 1 µg mL^–1^ for each compound, except for ERU, bDtBPP, BBOT, HEX, OLE, and STE, which were spiked at a final concentration of 100 ng mL^–1^, because of their limited solubility. After spiking, solutions were gently agitated for 20 min, followed by filtration through a 0.2 µm PES membrane (Fisher Scientific, Ireland) to remove any precipitation of the spiked compounds that may have occurred in the presence of the protein solution.

### UF/DF Process Operation: Setup and Sampling

An ÄKTA Flux S UF/DF system (Cytiva, UK) was used for this study with a 30 kDa Millipore Pellicon-3 cassette with Biomax^®^ membrane cassette (88 cm^2^ surface area and type A screen, catalogue number P3B030A00, Merck, Ireland). The full system preparation and setup for the UF/DF runs, determination of the NWP to verify membrane suitability, system equilibration, membrane passivation (to correct for non-specific binding impact of spiked species) and cleaning after process were performed as described in a previous study [26].

**Fig. 1:**
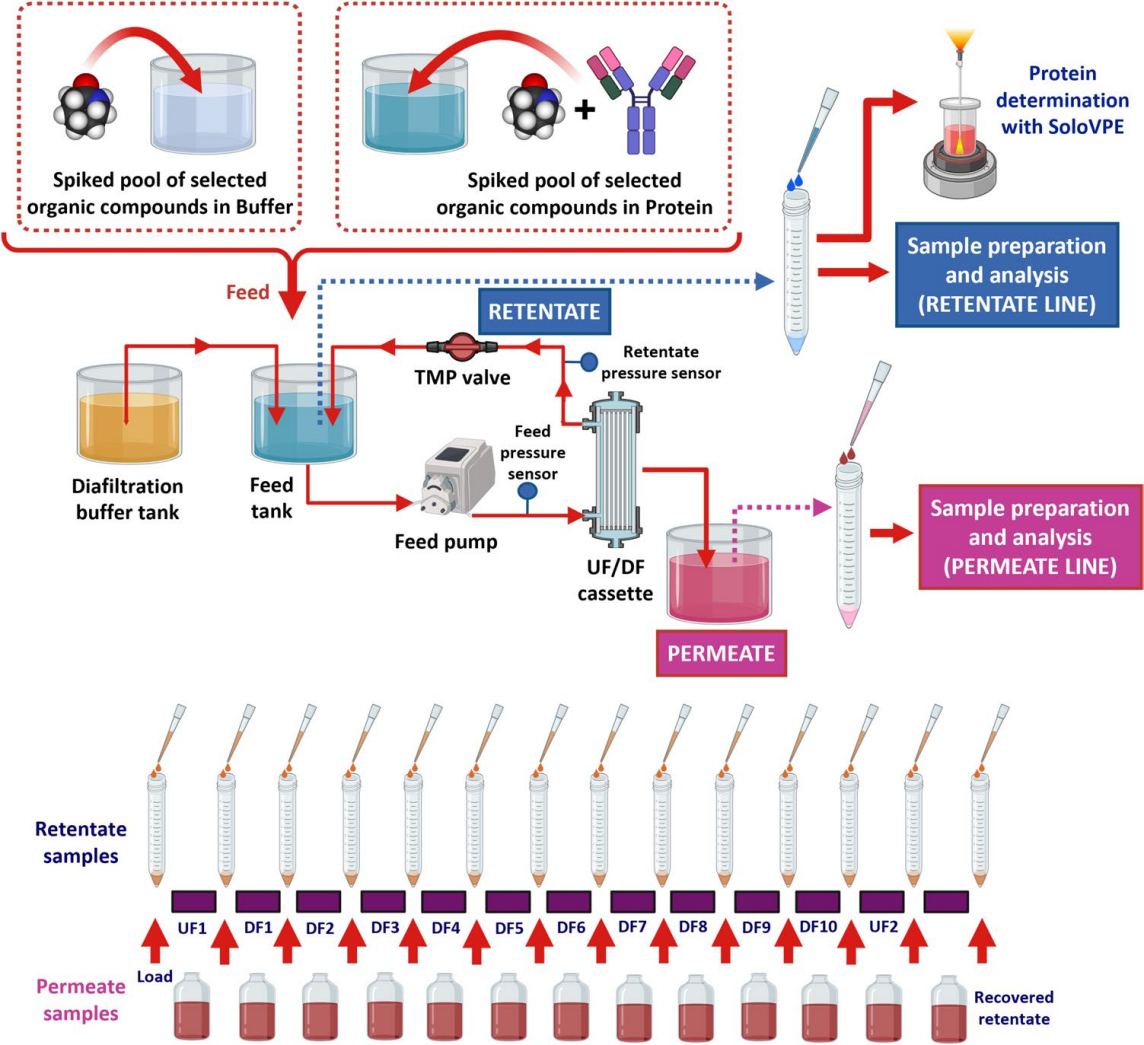
Schematic roadmap showing the full flow path of the UF/DF process, sampling and experimental setup. Figure was created using Biorender.com

Figure 1 displays the full flow path of the UF/DF process, sampling and experimental setup. The load protein solution was placed into the feed tank. The system was operated within the required TMP range for each process, consisting of an initial UF concentration step (UF1), followed by 10 DF steps, where the system volume was held constant (diavolume) and a final protein overconcentration step (UF2).

Table 1S shows the process conditions used for each protein. Process parameters were scaled down from the corresponding UF/DF commercial process. The UF/DF runs were carried out  in triplicate for each protein.

Retentate samples (2 × 500 µL) were taken from the feed tank in glass conical tubes, as shown in Fig. 1: from the initial protein load, after each process stage and from the final recovered retentate. Permeate samples from each step were collected in individual glass bottles. An additional 20 µL of sample were taken from the initial load and the final concentrated protein to measure its concentration and calculate protein recovery.

### Sample Preparation for Retentate Samples

**Fig. 2:**
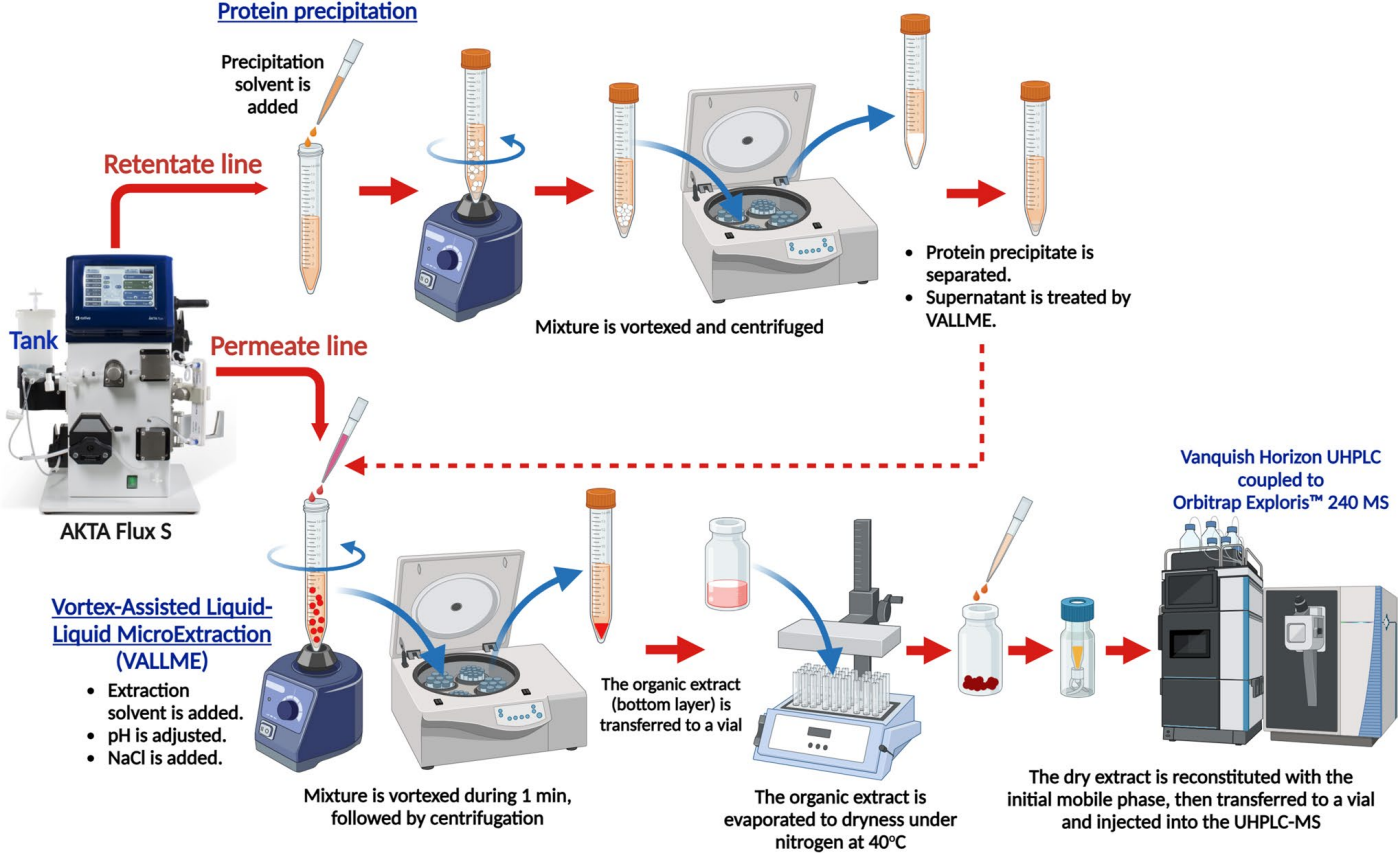
Analytical workflow for the determination of the selected organic compounds in retentate and permeate samples. Figure was created using Biorender.com

Collected retentate samples from each UF/DF step were processed simultaneously as shown in Fig. 2, for separate determination of compounds under positive and negative polarity. Deproteination was performed by protein precipitation carried out with an organic solvent optimized for each protein at a determined sample:solvent ratio, to ensure complete protein precipitation (Table 2S). Then, mixtures were vortexed for 1 min and centrifuged at 3,000 × *g* for 10 min. Supernatants were transferred to glass conical tubes for extraction (Sect. 2.5). Retentate samples from blank protein UF/DF runs were also processed following similar procedures.

### Sample Preparation Stage

Two permeate samples and deproteinated samples from each UF/DF step were extracted by VALLME (Fig. 2). Samples were prepared in glass conical tubes and adjusted to the extraction pH (Table 3S), followed by the addition of NaCl to a final concentration of 20% to adjust the ionic strength. If needed, the final sample volume was adjusted to 2 mL with deionized water. Then the extraction solvent, DCM, containing the internal standards at a concentration of 5 µL mL ^–1^ (Table 3S) was added to both samples. Mixtures were vortexed at the maximum speed (2,500 rpm) (Vortex 2 shaker, IKA, Germany) for 1 min. Afterwards, tubes were centrifuged at 3,000 × *g* for 10 min. The organic extracts (bottom layer) were transferred to small glass vials and evaporated to dryness under N_2_ at 40°C. Then, the two resulting dried extracts were reconstituted with 500 µL of the two initial mobile phases of the chromatographic methods: 0.2% (v/v) formic acid in water:MeOH (8:2), and 0.025% (v/v) NH_4_OH:MeOH (8:2) for further UHPLC-MS analysis under positive and negative polarity, respectively. Blanks using deionized water, equilibration, and diafiltration buffers were also processed for analysis.

### UHPLC-MS Analysis

UHPLC-MS analysis was performed using a Vanquish Horizon UHPLC system (Thermo Scientific, Germering, Germany), equipped with a binary pump-H, split autosampler-HT, and column compartment-H. The LC system was coupled to an Orbitrap Exploris 240 mass spectrometer with HESI interface (Thermo Scientific, Bremen, Germany). Parameters for chromatographic separation and for HRAM full-scan MS analysis collected in DDA mode are listed in Table II.

**Table II:** LC–MS Parameters

**LC parameters**	
Columns	Hypersil GOLD C8 100 × 2.1 mm, 1.9 µm (Positive polarity) Hypersil GOLD PFP 100 × 2.1 mm, 1.9 µm (Negative polarity)
Mobile phases	Positive polarity: Solvent A = 0.2% (v/v) formic acid in water; Solvent B = MeOH Negative polarity: Solvent A = 0.025% (v/v) NH_4_OH; Solvent B = MeOH
Flow rate	Constant at 0.4 mL min ^–1^
Column temperature	60 °C
Injection volume	5 µL
Gradient conditions	20% B (0–1.3 min), 20–98% B (1.3–12 min), 98% B (12–14 min), 98–20% B (14.0–14.1 min), and 20% B (14.1–18 min)
**MS parameters**	
Ion source parameters	
Ion source type	HESI
Spray voltage (positive ionisation), kV	3.5
Spray voltage (negative ionisation), kV	3.0
Source gas	High-purity N_2_
Sheath gas, a.u	50
Auxiliary gas, a.u	10
Sweep gas, a.u	1
Ion transfer tube temperature (°C)	320
Vaporiser temperature (°C)	330
Full-scan parameters	
Orbitrap resolution (at 200 m/z)	60,000
Scan range (m/z)	70–1,000
RF lens (%)	70
AGC target	1e^6^
Maximum spray current (µA)	100
Maximum injection time (ms)	100
Microscans	1
Data-Dependent Analysis Scan properties	
Top N	5
Isolation window (m/z)	1.2
Collision energy mode	Stepped
Collision energy type	Normalised
HCD collision energies (%)	20, 35, 50
HCD gas	Ultra-high-purity N_2_
Orbitrap resolution	30,000
AGC target	2e^4^
Maximum injection time (ms)	50
Microscans	1

^(*)^*PFP=* Pentafluoro-phenyl, *HESI* = Heated electrospray ionization, *HCD* = Higher energy collision induced dissociation, *AGC* - Automatic gain control, *RF*=  Radio frequency

For targeted analysis, full MS scan data allowed for screening and quantification of the selected organic compounds listed in Table I, based on the accurate masses of the targeted precursor ions. When operated in full MS/DDA mode, a product-ion spectrum with accurate mass measurement was obtained automatically according to precursor ions within a 10 ppm mass error window, which provided confirmation for the targeted compounds. Data acquisition and processing were performed using Chromeleon CDS Software version 7.3.2.

## Results and discussion

### Optimization of the Extraction Conditions by VALLME

Retentate samples after protein precipitation and permeate samples were extracted by VALLME. This method uses vortex agitation as a low-cost and effective way to break and disperse microliter drops of extraction solvent into the aqueous sample. The generation of fine droplets greatly increases the interfacial area available for mass transfer, reducing the diffusion distance and improving the extraction rates so that analytes can reach partition equilibrium quickly. Contrary to other extraction techniques, such as ultrasound- or microwave-assisted extraction, VALLME can supply the mechanical energy required for drop breakup. Moreover, emulsion formation in VALLME can proceed without adding a dispersive solvent, in contrast to DLLME [27]. After the extraction, the immiscible phases, the sample and the extraction solvent, were separated using centrifugation, and the acceptor phase was retrieved for analysis. In VALLME, analyte extraction predominately occurs during the first step of drop breakup (emulsion formation). Consequently, optimization of different extraction parameters were evaluated at this stage to improve extraction efficiency. The effect of extraction solvent (type and volume), sample volume, pH, and ionic strength were optimized for the selected organic compounds.

Vortex agitation time and speed are also critical parameters. High rotational speeds will increase Reynolds number and ensure a turbulent flow regime in the extraction tube during the emulsion formation step. At high agitation speeds, the size of droplets will be reduced, leading to an overall enhancement in extraction rates. Based on previous studies, vortex speed of 2500 rpm (maximum speed) for 60 s was selected as agitation conditions [28–30].

Selection of the most suitable extraction solvent is based on comparison of selectivity, extraction efficiency and aqueous solubility with respect to the target compounds. Chlorinated solvents are commonly selected as extraction solvents for DLLME and derived techniques [31], showing advantages, such as producing high enrichment factors, short extraction time, ease of operation and high sensitivity [30, 32].

Chloroform (CHCl_3_, $\rho$ = 1.47 g cm^−3^) and DCM (CH_2_Cl_2_,  $\rho$ = 1.32 g cm^−3^) were tested. Although chlorinated solvents usually have higher interfacial tension compared to other organic solvents [33], and more mechanical energy will be needed for the drop breakup, with the subsequent emulsion formation between sample and solvent, interfacial tension for chloroform and DCM are lower compared to other chlorinated solvents [34], ensuring a suitable emulsification process during extraction. In addition, these solvents also have low viscosities (0.54 and 0.42 mPa.s at 25 °C for chloroform and DCM, respectively), and, although the effect of the solvent viscosity on extraction efficiency is not thoroughly considered in VALLME, this parameter should affect the process of drop breakup, especially for viscous solvents, affecting the deformation process and leading to the formation of different sets of drop sizes with a different projected interfacial area, potentially impacting on extraction efficiency [35].

Figure 1S(a) shows that DCM was the solvent of choice, as it achieved higher extraction efficiency (measured as %recovery) for the targeted organic compounds compared to chloroform.

The volume of the extraction solvent (for 2 mL of sample), pH and ionic strength was optimized using a Box-Behnken design. The matrix consisted of 15 experiments including 3 centre points (Table 4S). Three levels for the variables were considered: solvent volume (from 40 to 250 µL), pH (from 2 to 10) and NaCl content (from 0 to 20%). Experimental data were evaluated by ANOVA which yielded R^2^ between 0.719 and 0.968. Since the P values for the lack-of-fit test were > 0.05 in all cases, the model appears to be satisfactory at the 95% confidence level.

Desirability plots were used to calculate the optimum solvent volume, pH and NaCl concentration, presented in Fig. 1S(c) and (d), for compounds analysed under positive and negative polarity, respectively.

Optimum solvent volumes were 125 µL (for positive polarity) and 100 µL (for negative polarity) for the extraction of 2 mL of sample. The use of low solvent to sample volume ratios is beneficial to improve method sensitivity [36], ensuring low collision frequencies and, as such, low recoalescence rates between droplets during emulsion formation [35]. This enhances the generation of smaller droplet sizes and increases the interfacial area available for analyte transfer.

Regarding pH optimization, pH was adjusted to favour the neutral form of analytes with acid–base chemistries, improving the extraction efficiency, as the neutral form may have a greater affinity for the extraction solvent [37]. pH 5 and 2 were the optimum for compounds determined under positive and negative polarity, respectively. Figure 1S(b) shows most compounds are in their neutral forms at the optimum pH values.

The influence of ionic strength on VALLME remains unclear but is anticipated to affect processes related to mass transfer, as it might alter the diffusion coefficients in the aqueous phase and the mass transfer at the interface due to modifications in the electrical double layer [38]. It is noted that the presence of salt may also affect emulsion stability [39] and sample viscosity [40]. The maximum tested NaCl concentration, 20%, was the optimum for both types of analytes, revealing a positive effect on extraction efficiency due to the salting out effect.

### Method Validation

The method developed for the determination of organic leachables in the three protein materials was validated according to the guideline ICH Q2(R1) “Validation of Analytical Procedures” [41]. The parameters evaluated were linearity, selectivity, sensitivity and accuracy (trueness and precision). Statistical parameters for the method are summarized in Table III.

**Table III:** Analytical and Statistical Parameters

Parameters	NDMA	NMP	CAP	NPIP	EMP	MBZ	DIB	ETS	6DTBP	BBS
Internal standard	TINP	TINP	TINP	TINP	TINP	BPA-d16	TINP	BPA-d16	BPA-d16	BPA-d16
Protein 1										
n	21	21	21	21	21	21	21	21	21	21
S_y/x_	1.85E-01	1.76E-01	1.13E-01	1.59E-01	1.23E-01	2.34E-01	9.20E-02	1.50E-02	1.66E-01	2.58E-01
b (mL ng^–1^)	3.40E-02	4.30E-02	2.30E-02	2.70E-02	1.70E-02	6.00E-02	2.30E-02	3.40E-03	2.00E-02	9.50E-02
S_b_ (mL ng^–1^)	9.70E-05	1.00E-04	6.50E-05	9.10E-05	7.10E-05	1.30E-04	5.30E-05	8.70E-06	9.60E-05	1.90E-04
R^2^ (%)	99.93	99.94	99.93	99.83	99.94	99.89	99.98	99.87	99.82	99.98
a	8.34E-03	5.60E-03	8.24E-03	−1.67E-03	6.60E-03	4.73E-03	6.00E-03	−6.89E-05	−2.83E-04	1.87E-02
S_a_	3.74E-02	3.52E-02	2.25E-02	3.18E-02	2.46E-02	4.67E-02	1.84E-02	3.02E-03	3.33E-02	6.56E-02
LOD (ng mL^–1^)	3.48	2.62	3.15	3.77	4.63	2.50	2.56	2.83	5.32	1.74
LOQ (ng mL^–1^)	11.61	8.74	10.49	12.57	15.45	8.33	8.54	9.42	17.72	5.80
Protein 2										
n	21	21	21	21	21	21	21	21	21	21
S_y/x_	2.50E-02	3.81E-01	1.20E + 00	1.14E-01	1.53E-01	2.80E-01	1.10E-01	2.25E-01	2.64E-01	1.60E-01
b (mL ng^–1^)	4.40E-03	6.50E-02	2.60E-01	2.00E-02	1.75E-02	4.70E-02	3.60E-02	5.60E-02	3.30E-02	4.50E-02
S_b_ (mL ng^–1^)	1.40E-05	2.20E-04	6.90E-04	6.50E-05	8.80E-05	1.60E-04	1.30E-04	2.00E-04	9.40E-05	9.20E-05
R^2^ (%)	99.96	99.94	99.92	99.92	99.87	99.94	99.94	99.95	99.97	99.82
a	2.10E-04	−1.80E-02	4.01E-02	5.70E-03	4.90E-03	−3.67E-03	1.88E-02	−6.88E-03	−1.72E-02	−1.37E-02
S_a_	5.01E-03	7.62E-02	2.41E-01	2.27E-02	3.07E-02	5.60E-02	4.65E-02	6.91E-02	3.28E-02	3.19E-02
LOD (ng mL^–1^)	3.64	3.75	2.96	3.65	5.60	3.82	1.97	2.57	5.12	2.28
LOQ (ng mL^–1^)	12.13	12.51	9.88	12.17	18.66	12.72	6.55	8.57	17.06	7.59
Protein 3										
n	21	21	21	21	21	21	21	21	21	21
S_y/x_	1.01E-01	1.41E-01	1.69E-01	1.04E-01	1.62E-01	9.19E-03	1.24E-01	1.63E-02	7.01E-02	4.47E-02
b (mL ng^–1^)	2.58E-02	1.85E-02	2.40E-02	2.02E-02	2.41E-02	2.06E-03	2.98E-02	3.46E-03	9.61E-03	1.40E-02
S_b_ (mL ng^–1^)	3.28E-04	3.04E-05	3.39E-05	5.82E-05	2.22E-05	3.04E-06	3.79E-05	4.08E-06	1.96E-05	3.24E-06
R^2^ (%)	99.88	99.79	99.74	99.11	99.89	99.72	99.79	99.82	98.87	99.65
a	6.94E-03	2.48E-03	1.15E-03	−5.02E-03	3.38E-03	−5.41E-04	−3.15E-03	−1.00E-04	2.25E-03	1.37E-04
S_a_	3.71E-04	1.55E-04	7.31E-04	2.97E-04	1.13E-04	1.55E-05	1.93E-04	2.08E-05	9.98E-04	1.65E-05
LOD (ng mL^–1^)	2.60	4.89	4.49	3.29	4.30	2.85	2.65	3.01	4.67	2.05
LOQ (ng mL^–1^)	8.66	16.31	14.98	10.97	14.34	9.51	8.83	10.04	15.57	6.83

Parameters	BHT	BPA	BHT-CHO	BHT-COOH	TTB	TBP	ERU	ATBC	BBOT	bDtBPP
Internal standard	BPA-d16	BPA-d16	BPA-d16	BPA-d16	BPA-d16	TINP	TINP	TINP	TINP	TINP
Protein 1										
n	21	21	21	21	21	21	21	21	21	21
S_y/x_	8.00E-02	5.40E-02	5.00E-02	2.81E-01	2.16E-01	2.55E-01	1.10E-01	2.03E-01	3.74E-01	3.73E-01
b (mL ng^–1^)	1.10E-02	9.80E-03	9.70E-03	5.60E-02	7.00E-02	9.50E-02	2.40E-02	9.00E-02	4.90E-02	9.30E-02
S_b_ (mL ng^–1^)	3.30E-05	3.70E-05	3.80E-05	1.60E-04	1.20E-04	1.50E-04	6.30E-05	1.30E-04	1.60E-04	2.40E-04
R^2^ (%)	99.97	99.93	99.9	99.92	99.97	99.94	99.94	99.93	99.98	99.94
a	5.87E-03	6.19E-03	1.78E-03	−6.42E-03	2.65E-03	−8.39E-03	9.06E-03	8.72E-03	1.76E-02	−1.60E-02
S_a_	1.15E-02	1.28E-02	1.33E-02	5.63E-02	4.32E-02	5.09E-02	2.20E-02	4.53E-02	5.48E-02	8.49E-02
LOD (ng mL^–1^)	4.65	3.53	3.30	3.21	1.98	1.72	2.94	1.44	4.89	2.57
LOQ (ng mL^–1^)	15.52	11.77	11.02	10.71	6.59	5.73	9.78	4.81	16.28	8.55
Protein 2										
n	21	21	21	21	21	21	21	21	21	21
S_y/x_	2.87E-01	3.12E-01	4.08E-01	4.10E-02	4.50E-02	9.28E-01	1.72E-01	1.88E-01	2.71E-01	1.60E-01
b (mL ng^–1^)	4.10E-02	9.00E-02	2.00E-02	4.20E-03	4.40E-03	2.20E-01	1.80E-02	1.00E-02	4.00E-02	1.34E-02
S_b_ (mL ng^–1^)	1.70E-04	2.40E-04	2.30E-04	2.30E-05	7.30E-05	5.30E-04	1.60E-04	1.10E-04	1.60E-04	7.50E-04
R^2^ (%)	99.94	99.96	99.97	99.9	99.99	99.98	99.85	99.93	99.98	99.89
a	−3.28E-03	1.09E-02	−2.00E-03	3.56E-03	−8.00E-03	−5.67E-02	1.40E-02	−1.29E-02	−2.79E-02	1.10E-02
S_a_	5.74E-02	8.23E-02	8.17E-02	8.15E-03	2.56E-02	1.86E-01	5.43E-02	3.77E-02	5.42E-02	1.69E-03
LOD (ng mL^–1^)	4.48	2.22	13.06	6.25	6.61	2.70	6.13	12.04	4.34	8.28
LOQ (ng mL^–1^)	14.95	7.41	43.55	20.84	22.03	9.00	20.44	40.14	14.46	27.61
Protein 3										
n	21	21	21	21	21	21	21	21	21	21
S_y/x_	9.56E-02	1.50E-01	9.94E-01	4.84E-02	2.68E-02	6.31E-01	3.91E-01	4.81E-01	5.52E-01	4.00E-02
b (mL ng^–1^)	1.05E-02	2.30E-02	6.41E-02	4.73E-03	3.23E-03	8.75E-02	3.80E-02	4.82E-02	5.87E-02	3.49E-03
S_b_ (mL ng^–1^)	1.13E-05	4.30E-06	4.73E-05	5.44E-06	6.29E-06	9.47E-05	5.73E-05	8.42E-05	4.78E-05	1.01E-05
R^2^ (%)	99.85	99.55	99.45	99.83	99.51	99.85	99.71	99.61	99.91	98.91
a	2.99E-03	−9.46E-04	−1.70E-03	−1.15E-04	−9.49E-04	3.46E-03	3.95E-03	2.01E-03	3.00E-03	1.07E-03
S_a_	5.74E-04	2.19E-05	8.83E-04	2.78E-05	3.21E-05	4.83E-04	2.92E-04	4.30E-04	2.44E-04	5.17E-04
LOD (ng mL^–1^)	5.80	4.17	9.93	6.54	5.32	4.61	6.59	6.38	6.02	7.34
LOQ (ng mL^–1^)	19.33	13.90	33.08	21.80	17.74	15.38	21.96	21.26	20.05	24.47

^(*)^*n* = points of calibration, *a =* intercept, *S*_*a*_ = intercept standard deviation, *b* = slope, *S*_*b*_ = slope standard deviation, *S*_*y/x*_ = regression standard deviation, *R*^*2*^ = determination coefficient, %, *LOD* = limit of detection, *LOQ* = limit of quantification

A matrix-matched calibration approach was used for the method validation, meaning standards containing the selected compounds were prepared in the protein formulations, being further analysed using the same procedure as the samples, this way the matrix effects during analysis were considered. The method showed good linearity within the concentration ranges with R^2^ ranging from 98.9% to 99.9% and P_lof_ values were higher than 5% in all cases.

Selectivity was determined by analysis of blanks and spiked blank samples containing the analytes. Retention times showed no interference with all compounds studied. High precision in retention times was also observed, with RSD values lower than 1%. Data confirm the high selectivity and retention time precision of the LC–MS method.

Method sensitivity was examined by the LOD (3.*s*_*0*_), and LOQ (10.*s*_*0*_), which were calculated using *S*_*y/x*_, *b* of the calibration plots and an estimate *s*_*0*_ obtained by extrapolation of the standard deviation of the blank. Experimentally determined LOQs ranged from 4.81 to 43.55 ng mL^−1^. Figure 2S(a) shows variation of LOQs per compound for the three protein materials. Pearson product-moment correlation coefficients established a positive correlation between LOQs and molecular weight (0.65) and a negative correlation with Log P (0.95) (p-values < 0.05 at 95% confidence level), showing that a higher sensitivity of MS-based methods is expected for polar low-molecular-weight compounds, which could be explained based on their higher ionization efficiencies. Differences were also observed in LOQs across the three-protein materials Fig. 2S(b). The method is more sensitive for P1 compared to P2 and P3, which have compounds with the highest LOQs. These differences could be potentially related to the buffer composition. Amino acids (arginine and/or histidine) present in buffers from P2 and P3 (Table 1S) could compete with the analytes under study for ionization in the HESI source, affecting method sensitivity. Sucrose seems not to produce an important impact, since it is present in buffers from P1 and P3 at similar concentrations.

The precision of the method in terms of intra- and inter-day variability was evaluated using spiked samples at three concentration levels (20, 200, and 800 ng mL^−1^) for each compound. Results are shown in Table 5S. Precision, expressed as %RSD, was determined from triplicate spiked samples analyzed on the same day (repeatability between 0.1% and 6.9%) and across 7 different days (reproducibility between 0.1% and 7.2%).

Due to the absence of Certified Reference Materials, relative recovery assays were performed to validate the trueness of the method, with results presented in Table 5S. Recoveries were determined by analysing spiked blank samples and the concentration of each compound was determined by interpolation using the standard calibration curve within the linear dynamic range and compared with the known concentration of the analytes spiked into the samples. The relative recoveries were close to 100% (98.0% to 104.7%) in all cases.

Precision and trueness data indicated that the method is highly reproducible and robust, with similar performance for the three proteins.

### Mass Recovery Verification

**Fig. 3:**
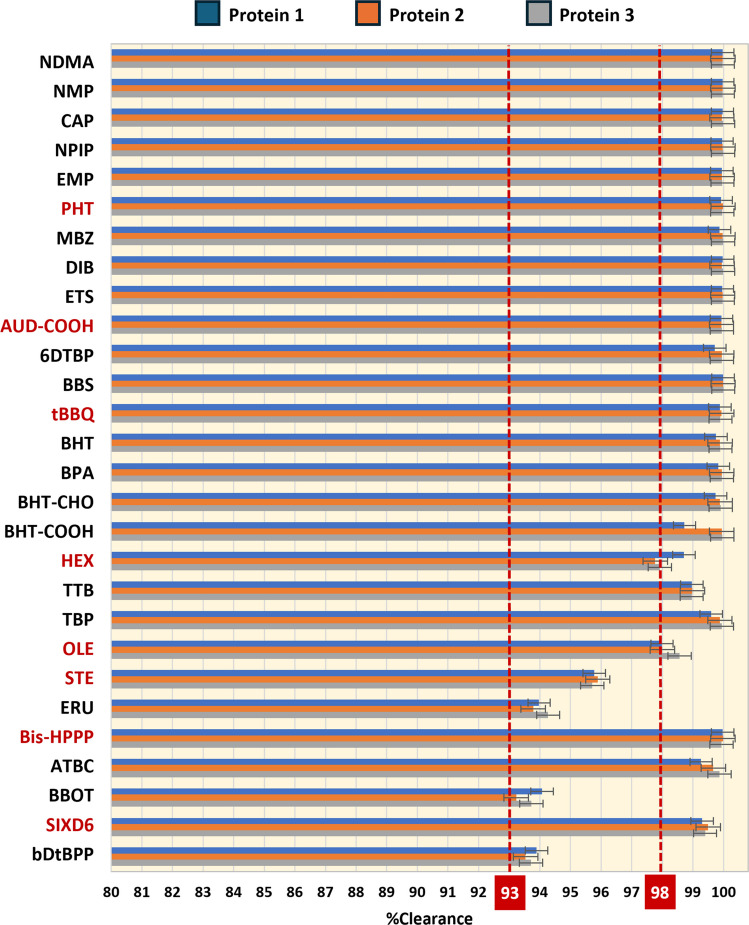
Removal percentages of selected organic compounds after 10 DVs for the 3 protein materials evaluated. Compounds from the prediction set are highlighted in red. Compounds were sorted by molecular weight

Investigation of clearance of organic compounds is only pertinent when UF/DF processes show a good mass balance (*i.e.*, the ratio of the sum of analyte amount in all permeate and recovered retentate fractions to the analyte amount introduced initially into the UF/DF system). Figure 3S compiles the percent mass recoveries for the runs with spiked buffer and spiked protein for the 3 protein materials. Mass balances between 85 and 114% were found, being in the range used as an acceptance criterion for further calculation of clearance parameters (75–125%) [42].

### Clearance of Organic Compounds through UF/DF

Organic compounds from different chemical classes with a wide range of MW and polarity were selected (Table I). The selection was based on their presence in representative biomanufacturing processes and E&L assessments from SUTs, as well as frequency of their occurrence in the processes [4]. In addition, only commercially available organic compounds were chosen, since their quantitation was required as part of the study. The protein materials were selected to include the range of isoelectric points for mAbs that are manufactured, so that the effect of the protein on clearance could be also evaluated.

Of the 28 compounds in total that were tested, 14 showed clearance over 99.9% and 10 were removed between 98% and 99.9%, on average for the three protein materials (Fig. 3). Only 4 compounds (STE, ERU, BBOT and bDtBPP), characterized by their high MW, showed slightly lower clearance, between 93 and 98%.

**Fig. 4:**
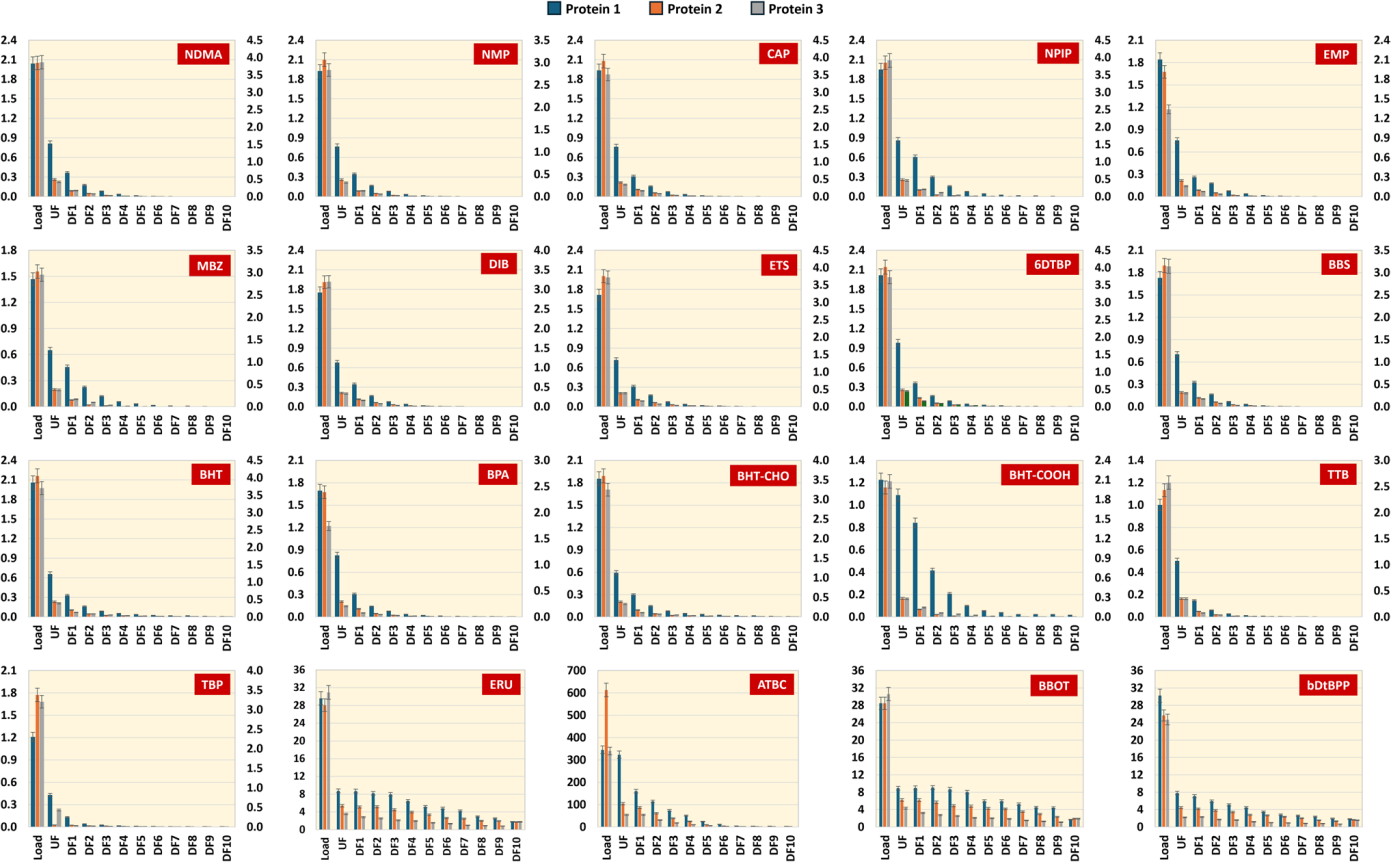
Clearance trends of the selected organic compounds along UF/DF processes for the 3 protein materials evaluated. The Y axis represents the mass of each compound in micrograms (primary axis = P1 and P3; secondary axis = P2). For ERU, ATBC, BBOT and bDtBPP: Y axis = P1, P2 and P3

A deeper evaluation of the clearance was performed through the determination of sieving coefficients for the selected organic compounds. During the UF/DF process, clearance of compounds occurs during both, UF and DF steps. In ultrafiltration, clearance is measured by the amount of leachables remaining in the retentate compared to the initial concentration. Leachable removal during DF follows an exponential trend as described by the Eq. (1):

1 $$C={C}_{0}\times {e}^{S\cdot N}$$

where C is the retentate concentration of the compound after a certain number of DVs, C_0_ is the concentration of the compound at the beginning of DF, *N* is the number of DVs, and *S* is the sieving coefficient of the compound under the evaluated process conditions [43]. A sieving coefficient of 1 signifies that substances pass freely through the membrane to the permeate side, whereas a value of S < 1 suggests that there is some degree of retention of compounds on the retentate side of the membrane. Figure 4 confirmed that clearance of compounds followed an exponential reduction leading to ideal removal for most of the spiked molecules, supporting trends observed in Fig. 3.

To determine the sieving coefficients for each compound, the natural logarithm of the concentration of each was plotted against the number of DVs, and the resulting negative slope from the linear regression represents the sieving coefficient, shown by Eq. (2):

2 $$\mathrm{ln}C=\mathrm{ln}{C}_{0}-S\cdot N$$

Table 6S shows the sieving coefficients from retentate solutions for the organic compounds studied, calculated from the slopes of the linear regression plots.

**Fig. 5:**
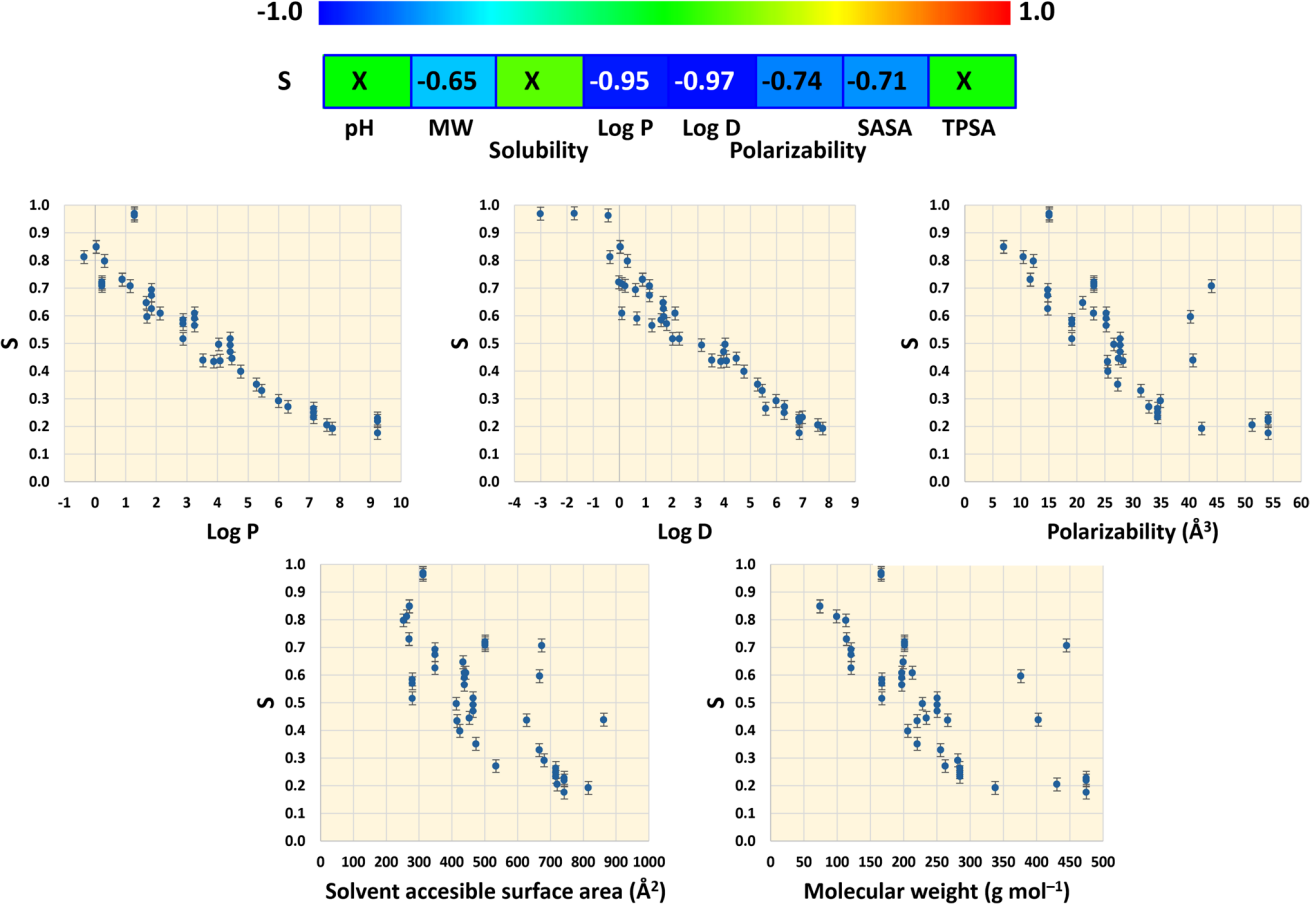
Pearson product-moment correlations between the sieving coefficient and relevant physicochemical properties of organic compounds. Correlation coefficients range between –1 and + 1 and measure the strength of the linear relationship between the variables. Coefficients are shown only when P-values are below 0.05, indicating statistically significant correlations at 95% confidence level. “X” represents no significant correlation at the 95% confidence level. Properties for the compounds were obtained from Chemicalize (Chemaxon.com)

Pearson product-moment correlations were measured to identify parameters that impacted clearance, either from UF/DF process conditions or physicochemical properties of the compounds. Figure 5 shows that Log P and Log D are the most critical properties that influence sieving coefficients. Log P is the logarithm of the 1-octanol/water partition coefficient, which has been used as a measure of lipophilicity [44]. Log P is used as a general standard property [45], which describes the partition of unionized compounds between two immiscible phases (1-octanol/water). Log P can provide useful information about other properties, such as water solubility, and represents an indirect measurement of polarity. In general, Log P < 1 indicates high solubility in water (high polarity), while Log P > 3 represents high solubility in nonpolar solvents (low polarity). In the presence of a basic or acidic group, the ionization of a molecule provides an additional factor to consider, since the partition then becomes pH dependent, being measured by Log D, which looks at the differential distribution of charged and uncharged forms of the molecule [46].

The negative correlation between sieving coefficients and Log P and Log D indicates that polar compounds will be more efficiently cleared during UF/DF. However, since Fig. 5 also shows that process pH is not an influential parameter, distribution of charged forms of the molecules is not a determining factor in their clearance.

MW also shows a significant correlation with sieving coefficients. Larger molecules are more likely to be less polar (higher Log P), explaining the negative correlation. Likewise, the negative Pearson coefficient for polarizability is mostly related to the MW, since heavier molecules are usually larger and more polarizable. The impact of the SASA is associated with the structure of the molecule and the presence of functional groups that interact with the solvent.

Examples that demonstrate the relevance of Log P over other properties are the clearance behaviour of ATBC, SIXD6, and Bis-HPPP. They belong to the group of compounds with the highest MW (over 300). However, their clearance percentage and sieving coefficients are like those of compounds with lower MW (Fig. 3). Despite their high MW, ATBC, SIXD6 and Bis-HPP have Log P of 3.53, 1.15 and 1.70 respectively compared to Log P values of ERU, BBOT and bDtBPP that are higher than 7. The lower Log P of these compounds can be explained by the presence of multiple polar groups (Fig. 4S), which dominate over the hydrophobic character of the alkyl residues represented by the MW. In addition, some of these compounds also show lower polarizability and higher TPSA, than compounds with similar high MW. These compounds clearly show the notable relevance of Log P over MW and other properties in explaining clearance behaviour, also established by the Pearson coefficients (Fig. 5).

UF/DF process conditions showed a minimal impact on clearance of the tested compounds in the three protein materials under normal processing conditions, with similar protein recoveries ranging from 94 to 98%. RSD values for percentages of clearance and sieving coefficients of the studied compounds across the three protein materials were lower than 0.7% and 1%, respectively.

As previously mentioned, sieving coefficients are independent of the process pH. This finding is supported by a previous study about clearance of solvents and small molecules in UF/DF processes of antibody–drug conjugates [21]. The irrelevance of process pH on clearance of organic leachables is confirmed by the compounds EMP, PHT, MBZ, DIB, AUD-COOH, BHT-COOH, STE and bDtBPP, whose sieving coefficients were not impacted, despite having different Log D values and charges under the three process conditions.

One challenge associated with high concentration mAb formulations resulting from UF/DF processes is the increased electrostatic interaction between proteins and small molecules. Such interactions may lead to an offset between the amount of leachables in the final products and diafiltration buffers used. The influence of such electrostatic interactions in a membrane process is known as the Gibbs-Donnan effect [47], which controls diffusion of charged species across the membrane. Consequently, the Gibbs-Donnan effect can influence removal of leachables, based on the electrostatic interaction between the charged protein and charged leachables.

Most compounds were uncharged under the process conditions, with only 7 having a net charge: positive for EMP and DIB, and negative for MBZ, BHT-COOH, PHT, STE and bDtBPP. The protein materials exhibited a net positive charge during the UF/DF processes, leading to a significant repulsion with positively charged compounds, thus hindering their interaction. Therefore, the removal efficiency is expected to be enhanced for compounds with the same charge as the protein and reduced for compounds with the opposite charge [48, 49]. However, similar clearance was observed between compounds with positive charge and uncharged, and a slightly reduced removal percentage was only observed for bDtBPP $\left(\sim 94\%\right)$ and STE $\left(\sim 96\%\right)$. Therefore, although Gibbs-Donnan effect could influence clearance, it does not dominate over other factors like Log P or MW, for instance.

### Modelling Approaches for Classification and Prediction Abilities

Data sets from UF/DF processes for the three protein materials were imported to SIMCA software version 17.0.1 (Sartorius, Sweden) for multivariate statistical analysis. PCA was used to identify outliers and, examination of the scores and loadings plot led to a better understanding of the different sources of variation in the data.

**Fig. 6:**
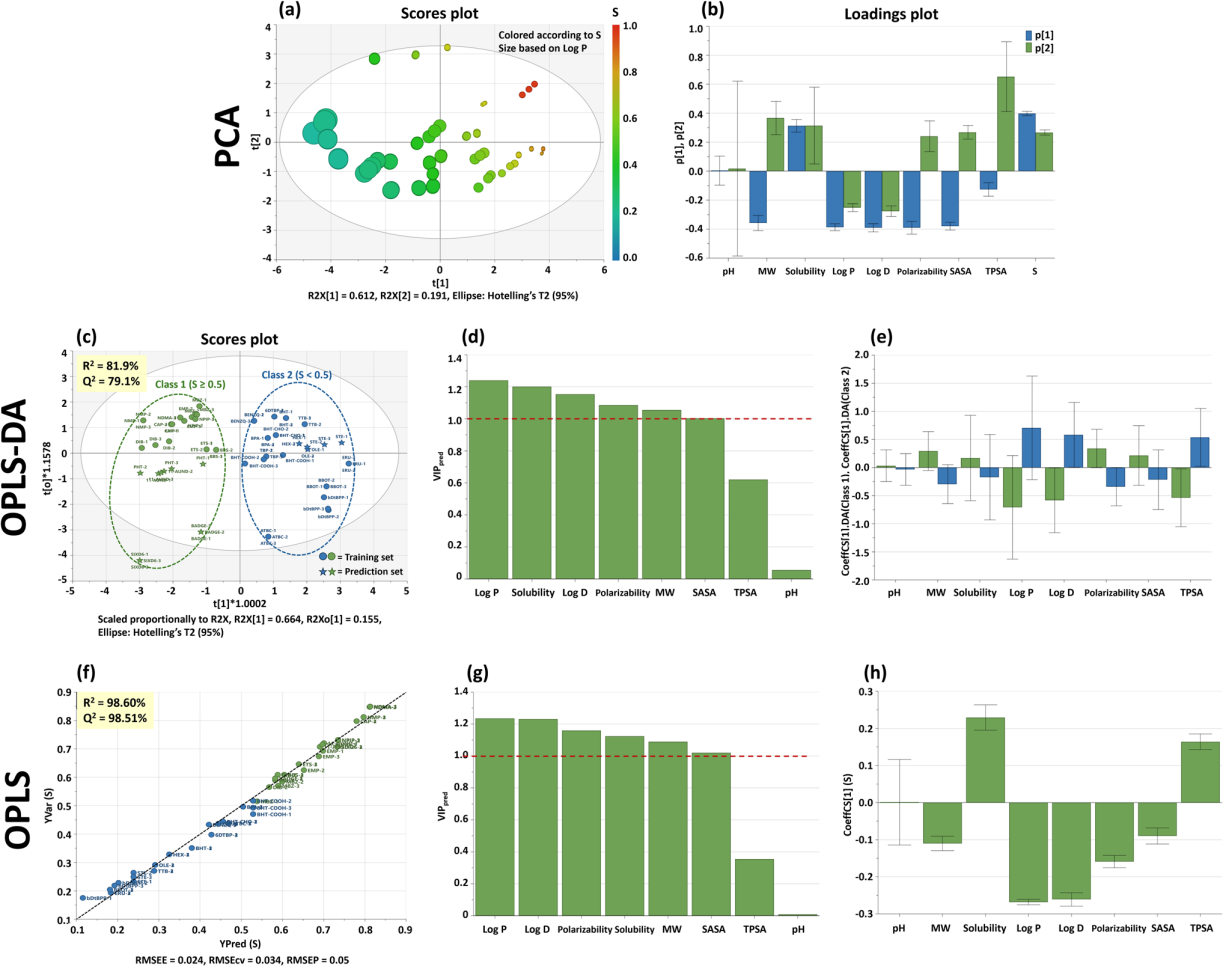
Multivariate analysis of the clearance data. PCA: Scores (**a**) and loadings (**b**) plots of PC 1 and PC2, representing 80.3% of the total variability of the data, and showing the influence of the indicated properties. OPLS-DA: compounds were classified in 2 groups based on sieving coefficients, shown in the scores plot for the training and prediction sets (**c**). Plots for VIP indicators (**d**), and regression coefficients (**e**) are also presented. OPLS: Predictions on sieving coefficients for the training and prediction sets (**f**). Plots for VIP indicators (**g**), and regression coefficients (**h**) for the model are also displayed

Figure 6(a) shows the scores plot of PC1 and PC2, which represent 80.3% of data variability. PC1 contains information about Log P, with the size of the data point corresponding to the magnitude of the Log P value. Log P decreases to the right along X-axis and is correlated with increasing sieving coefficients (indicated by the colour gradient). This correlation is highly significant, as it represents 61.2% of the data variability. From the loadings plot in Fig. 6(b), PC1 also comprises other properties such as Log D, MW, polarizability and SASA. PC2 containing 19.1% of data variability is mostly represented by TPSA. The loadings plot confirmed that pH does not significantly impact compound clearance.

Based on the PCA results, a supervised approach, OPLS-DA, was applied to a training set of 20 compounds (Table I) to model differences in clearance based on sieving coefficients, using physicochemical properties of the compounds. Experimental sieving coefficients were grouped in 2 classes: Class 1 compounds with sieving coefficients ≥ 0.5, and Class 2 compounds that showed lower clearance levels, with sieving coefficients < 0.5.

The OPLS-DA model was cross-validated in seven rounds by the leave-one-out method, yielding satisfactory values for the quality parameters, R^2^ and Q^2^ of 81.9% and 79.1%, respectively. The properties that had a significant influence on class discrimination were determined from the VIP plot, Fig. 6(d), which calculates the importance of each variable in the projection in the model, acting as an indicator for statistical refinement of the selection of properties (x-block). Terms with VIP larger than 1 are the most relevant for the class discrimination. Therefore, Log P, solubility, polarizability, MW and SASA were selected to categorize compounds in Class 1 or 2. TPSA and pH were dismissed in the final model because their VIP were significantly lower than 1. Log D was also excluded, because pH is not a decisive parameter. Consequently, removal of Log D did not impact on model performance and reduced the risk of overfitting, since the contribution of Log P is equivalent and sufficient. Figure 6(e) shows the regression coefficients associated with each class, confirming the correlations established previously with the Pearson coefficients and reinforcing the irrelevance of pH in determining compound clearance.

A prediction set of 8 compounds was selected to test the OPLS-DA model (Table I). UF/DF runs for the prediction set were carried out with the three proteins under the same conditions as for the training set. Figure 6(c) shows that all compounds from the prediction set were correctly grouped based on the sieving coefficients determined experimentally, Table 6S(b).

An OPLS model to forecast sieving coefficients (Y variable) was also developed from data, based on different properties of the compounds (x variables). The selection of relevant properties (x-block) will impact on model quality. Statistical approaches were employed to minimize the error between predicted Y and measured Y, including calculation of the error of the estimate, RMSEE, where Y predicted is calculated from the same training data that are used to make the model; the error of cross-validation, RMSE_CV_, and the error of prediction, RMSEP, that is calculated when the model is applied to the prediction set only. The OPLS model generated had a R^2^ of 98.6%, a Q^2^ of 98.5%, and a prediction error of 0.05 for the sieving coefficients, Fig. 6(f). The VIP plot was also used to select Log P, solubility, polarizability, MW and SASA, as properties to predict the sieving coefficient, Fig. 6(g). In addition, regression coefficients from Fig. 6(h) confirmed, once again, the correlations shown by the Pearson coefficients. Table 7S shows a summary of the model parameters presented in Fig. 6.

**Fig. 7:**
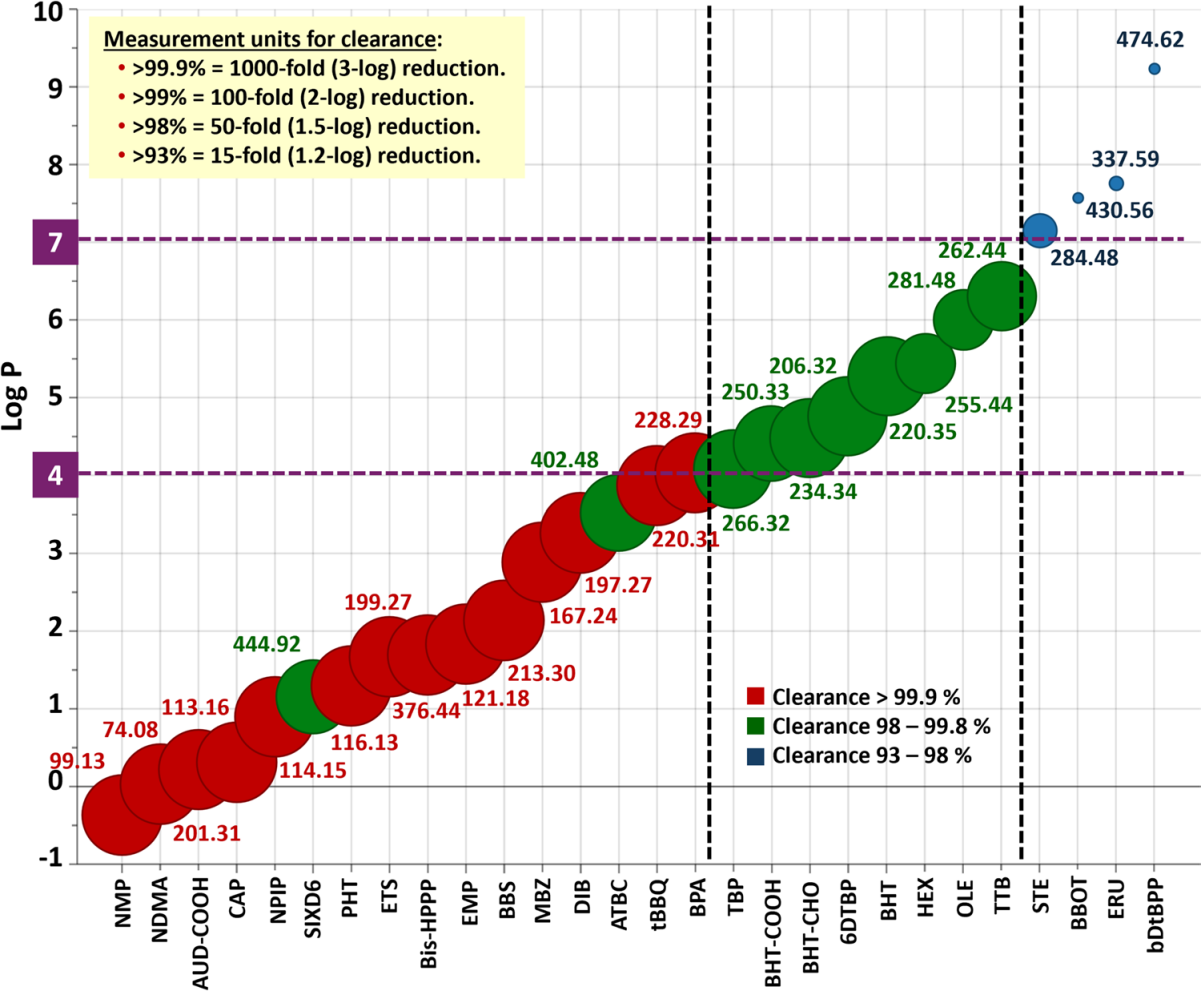
Summary of the clearance trends observed for the leachables under study and their correlations with the sieving coefficient, Log P, and MW. Size of the dot represents the average of the clearance percentage from the 3 proteins, which is also presented in 3 coloured ranges. Numbers on each dot correspond to MW. Equivalences among different measurement units for clearance are also displayed

Results presented in above have grouped compounds generally characterized by their clearance (Fig. 7 and Table 8S):

(i) Sieving coefficients close to ideal, with removal over 99.9% for compounds with Log P < 4;
(ii) Compounds with Log P between 4 and 7 achieved 98–99.9% removal with sieving coefficients between 0.27 to 0.5.
(iii) Lower clearance (93–98%) for compounds with Log P > 7, with sieving coefficients lower than 0.25, and MW over 280.

It is important to remark that although these are general trends, particular behaviour may be observed for some compounds, such as ATBC, Bis-HPPP and SIXD6, as discussed above.

Finally, although PERLs with high Log P (> 7) have lower clearance, the risk of entering the process or persisting into the final drug product above patient safety concern thresholds is low, considering their poor solubility in bioprocessing solutions (mostly aqueous based), which agrees with previous studies [19, 42]. Therefore, although highly hydrophobic PERLs may not be fully cleared, their removal over 93% is still good indication that UF/DF results in significant clearance of these compounds.

## Conclusions

This study established that UF/DF efficiently removed selected organic compounds, mostly additives and their degradation products, from SUTs used in previous manufacturing steps. A novel analytical method based on VALLME was developed for the analysis of retentate and permeate samples taken from UF/DF processes for three different proteins. Removal percentages were > 98% for 24 of 28 compounds. This study also demonstrated that process variables such as protein pI and concentration, buffer formulation, pH, membrane loading, TMP and cross flow rates had a minimal impact on clearance, so that sieving coefficients and removal percentages were similar for the three protein materials. Physicochemical parameters of the compounds were decisive to understand clearance. Log P was the most important factor. Compounds with Log P < 4 had generally sieving coefficients close to ideal clearance and over 99.9% removal. Compounds with Log P between 4 and 7 achieved 98–99.9% clearance, and compounds with Log P > 7 (STE, ERU, BBOT and bDtBPP) showed lower but still significant clearance with > 93% removal. Other influential parameters on clearance were polarizability, solvent accessible surface area and molecular weight.

Mathematical models utilizing OPLS regression were generated to categorize and forecast the clearance of organic compounds throughout UF/DF processes. A discriminant analysis model allowed classification of compounds to differentiate the ones with clearance close to ideal (> 99.9%) from the ones with lower but still significant clearance (> 93%). A second regression model was generated to predict sieving coefficients. Both models provide platforms that may aid industry and regulatory bodies to develop sophisticated risk-based PERL testing strategies. Notwithstanding these comments, the results documented in this study show that UF/DF presents significant risk reduction with respect to potential leachables arising from drug substance manufacturing processes including those arising from the UFDF circuit itself.

## Supplementary Information

Below is the link to the electronic supplementary material.

Supplementary file1 (PDF 1560 KB)

## Data Availability

Data available upon request.

## Abbreviations

ACN: Acetonitrile
AGC: Automatic gain control
*b*: Slope
bDtBPP: Bis(2,4-di-tert-butylphenyl)phosphate
CDS: Chromatography Data System
DCM: Dichloromethane
DDA: Data-dependent acquisition
DLLME: Dispersive liquid–liquid microextraction
DV: Diavolume
E&Ls: Extractables and leachables
FDA: U.S. Food and Drug Administration
HCD: Higher-energy collisional dissociation
HESI: Heated electrospray ionization
HRAM: High resolution accurate mass
LC-HRMS: Liquid chromatography high resolution mass spectrometry
LOD: Limit of detection
Log D: Distribution coefficient that depends on pH
Log P: Octanol-water partition coefficient
LOQ: Limit of quantification
mAb: Monoclonal antibody
MeOH: Methanol
MW: Molecular weight
NH4OH: Ammonium hydroxide solution in water
NWP: Normalized water permeability
OPLS: Orthogonal Partial Least Squares
OPLS-DA: Orthogonal partial least squares-discriminant analysis
P1: Protein 1
P2: Protein 2
P3: Protein 3
PC1: Principal component 1
PC2: Principal component 2
PCA: Principal component analysis
PERL: Process equipment related leachable
PES: Polyethersulfone
pI: Isoelectric point
P_lof_: Lack‐of‐fit test
Q^2^: Goodness of prediction
R^2^: Correlation coefficient
RMSE_CV_: Root mean square error of cross validation
RMSEE: Root mean square error of estimation
RMSEP: Root mean square error of prediction
RSD: Relative standard deviation
*S*: Sieving coefficient
SASA: Solvent accessible surface area
SUTs: Single‐use technologies
*S*_*y*__*/x*_: Standard deviation of the residuals
TMP: Transmembrane pressure
TPSA: Topological polar surface area
UF/DF: Ultrafiltration/diafiltration
UHPLC-MS: Ultra high performance liquid chromatography-mass spectrometry
VALLME: Vortex-assisted liquid–liquid microextraction
VIN: Post virus inactivation
VIP: Variable importance in the projection
