# Supplementary material for: Investigation into Clearance of Organic Compounds from Biomanufacturing Process Streams during Ultrafiltration/Diafiltration
Source: Pharm Res. 2026 Mar 24;43(4):1271–89. doi: 10.1007/s11095-026-04050-2 (PMC13179207; doi:10.1007/s11095-026-04050-2)
Supplement: Supplementary file 1 — Supplementary file1 (PDF 1560 KB) [file 11095_2026_4050_MOESM1_ESM.pdf]

Investigation into Clearance of Organic Compounds from Biomanufacturing Process Streams during Ultrafiltration/Diafiltration

Noemí Dorival-García[a], Anna Mulligan[a,e], Ronan Hayes[b], Charles Felice[c], Aidan Sexton[d], Ping-Ping Wang[c], and Jonathan Bones[a,e]

[a] Characterisation and Comparability Laboratory, The National Institute for Bioprocessing Research and Training (NIBRT), Foster Avenue, Mount Merrion, A94 X099, Co. Dublin, Ireland.
[b] Manufacturing Science and Technology, Johnson & Johnson Innovative Medicine, Barnahely, P43 FA46, Co. Cork, Ireland.
[c] Material Sciences at Discovery, Product Development & Supply, Johnson & Johnson Innovative Medicine, 200 Great Valley Parkway, Malvern, Pennsylvania 19355, United States.
[d] Material Sciences at Biotherapeutics Development & Supply, Johnson & Johnson Innovative Medicine, Barnahely, P43 FA46, Co. Cork, Ireland.
[e] School of Chemical and Bioprocess Engineering, University College Dublin, Belfield, Dublin 4, D04 V1W8, Ireland

Noemí Dorival-García: Noemi.DorivalGarcia@nibrt.ie
Anna Mulligan: Anna.Mulligan@nibrt.ie
Ronan Hayes: rhayes10@its.jnj.com
Charles Felice: cfelice@its.jnj.com
Aidan Sexton: asexton@ITS.JNJ.COM
Ping-Ping Wang: pwang9@its.jnj.com
Jonathan Bones: jonathan.bones@nibrt.ie (Corresponding Author)

Supporting information

Table 1S

UF/DF process range conditions

	P1	P2	P3
Protein pI	8.35	7.2	8.94
UF/DF conditions			
Equilibration buffer	Sodium acetate + NaCl pH 4.7	Histidine + Arginine pH 6.5	Sodium acetate + NaCl pH 5.5
Diafiltration buffer	Sodium acetate + sucrose pH 4.8	Histidine + Arginine pH 6.5	Histidine + sucrose pH 5.6
Concentration, g L^{-1} (volume, mL)			
Initial [(1)]	15.6 (200)	10 (390)	12.0 (200)
After UF1 [(2)]	30.0 – 55.0 (80)	Max. 90 (50)	92 – 108 (24)
After UF2 [(2)]	120 – 130 (60)	Max. 180 (25)	117 – 140 (20)
Diavolume (mL)	80	50	24

(1) Initial concentration of the load protein prior the UF/DF.

(2) Ranges correspond to the MOR (maximum operation range).

Table 2S

Protocol for protein precipitation of retentate samples

	P1	P2	P3
Sample volume (μL)	500	500	500
Precipitating solvent	ACN	Methanol	Acetone
Solvent volume (mL)	0.5	2.0	1.5
Ratio sample:solvent	1:1	1:4	1:3

Table 3S

Conditions for VALLME extraction of the samples

Extraction parameter	Positive	Negative
pH	5	2
Final NaCl (%)	20	20
Extractant volume (DCM, μL)	125	100

Figure 1S

Optimization of extraction parameters

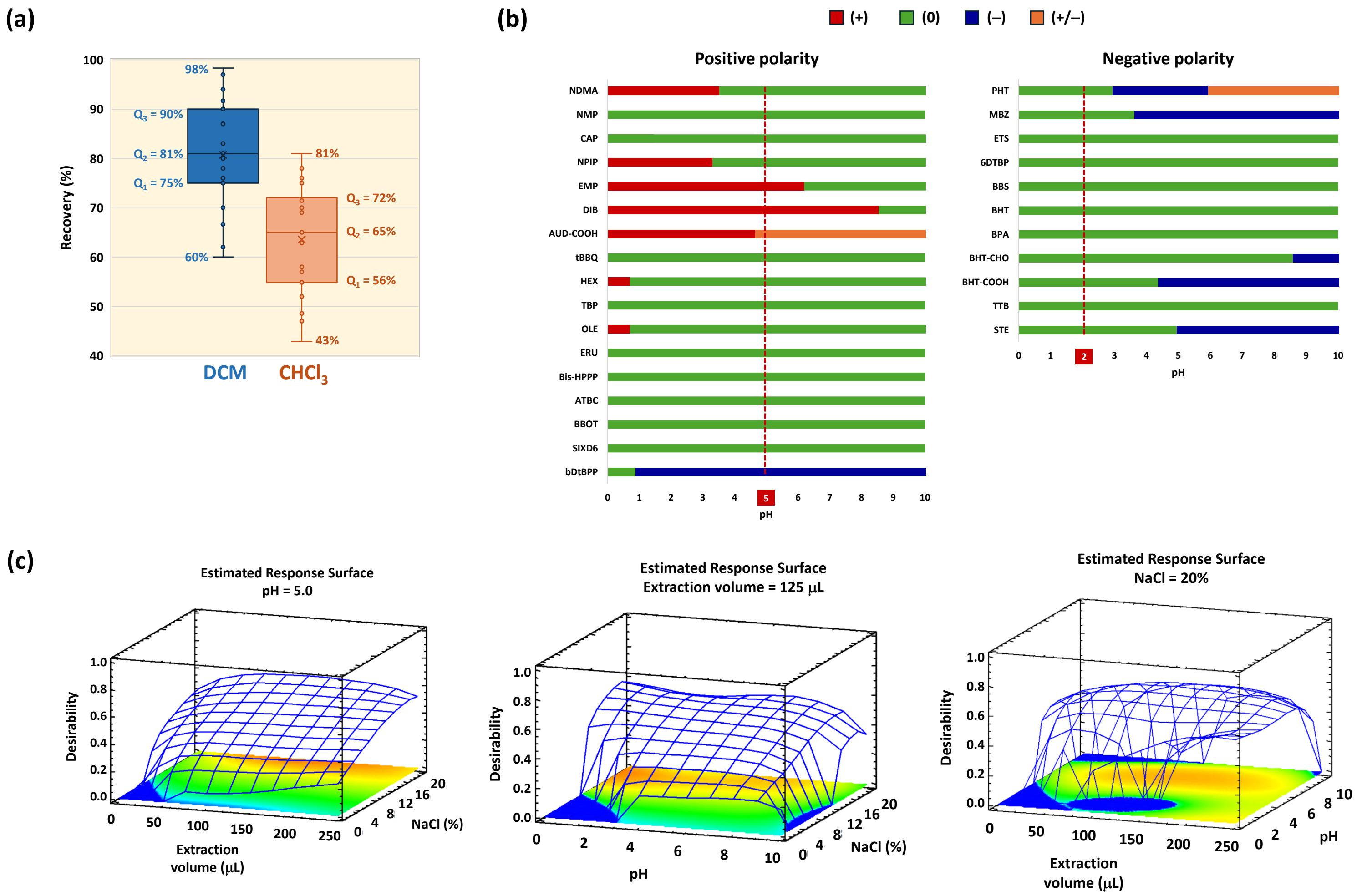

For compounds determined under positive polarity: Desirability = 0.62. Optimum conditions: pH 5.0; Volume of DCM = 125 μL (for 2 mL sample); NaCl = 20%.

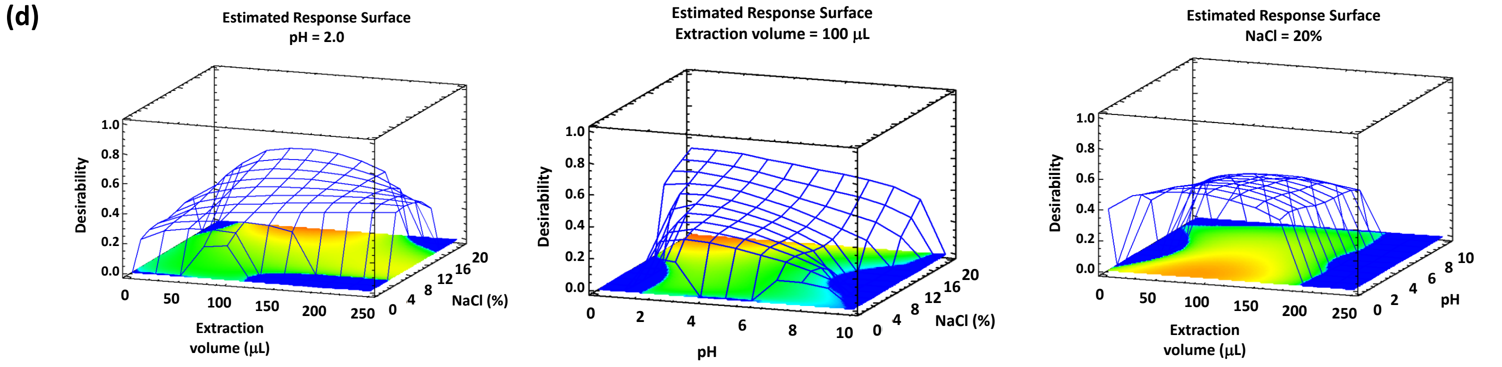

For compounds determined under negative polarity: Desirability = 0.53. Optimum conditions: pH 2.0; Volume of DCM = 100 μL (for 2 mL sample); NaCl = 20%.

Table 4S

Box-Behnken matrix for optimisation of solvent volume, pH and ionic strength.

Experiment	Extraction volume (uL)	pH	NaCl (%)
**1**	40	6	20
**2**	250	6	0
**3**	250	6	20
**4**	145	2	20
**5**	145	6	10
**6**	250	2	10
**7**	40	6	0
**8**	40	2	10
**9**	250	10	10
**10**	145	2	0
**11**	145	10	20
**12**	145	6	10
**13**	145	10	0
**14**	145	6	10
**15**	40	10	10

(*) In grey = centre points

Figure 2S

Limits of Detection (LODs) and Limits of Quantification (LOQs) of the analytical methods to determine leachables developed for the 3 protein materials.

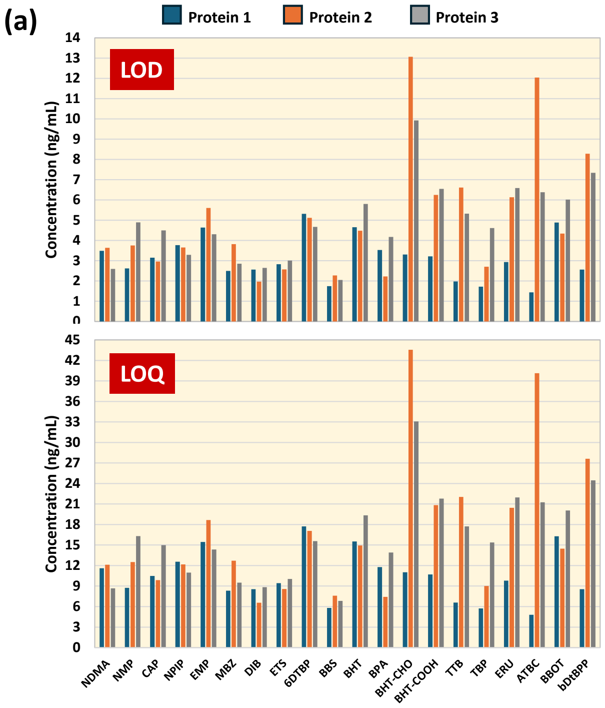

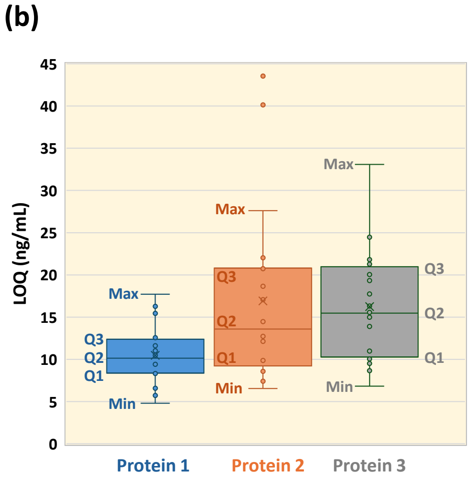

	Protein 1	Protein 2	Protein 3
Min	4.81	6.55	6.83
Max	17.72	43.55	33.08
Q1	8.49	9.66	10.74
Q2	10.14	13.59	15.47
Q3	11.97	20.76	20.35

Min and max or "whiskers" extend from the minimum value (Min) to the first quartile (Q1) and from the third quartile (Q3) to the maximum value (Max). They signify the range of the data, excluding potential outliers, and including the "box", that goes from Q1 to Q3. This box contains the middle 50% of the data and represents the interquartile range.

Table 5S

Method validation: Accuracy of the method. Precision and trueness of target compounds in samples.

Protein 1					Protein 2					Protein 3				
Compound	Spiked level ($ng\ mL^{-1}$)	Recovery (%)	Precision (%)		Compound	Spiked level ($ng\ mL^{-1}$)	Recovery (%)	Precision (%)		Compound	Spiked level ($ng\ mL^{-1}$)	Recovery (%)	Precision (%)	
			Intra-day	Inter-day				Intra-day	Inter-day				Intra-day	Inter-day
NDMA	20	99.9	3.2	5.7	NDMA	20	100.0	2.6	3.3	NDMA	20	100.1	3.8	0.7
	200	102.1	3.4	2.6		200	101.2	1.1	3.5		200	100.9	1.8	2.4
	800	98.7	0.8	1.1		800	101.7	2.4	3.4		800	98.1	0.1	1.6
NMP	20	102.9	0.6	3.0	NMP	20	101.2	1.6	0.6	NMP	20	100.4	0.3	0.2
	200	98.5	0.1	1.5		200	99.0	0.2	0.6		200	98.8	0.4	0.5
	800	98.8	0.6	0.8		800	99.3	0.9	0.1		800	99.4	0.2	0.3
CAP	20	103.3	4.6	1.4	CAP	20	100.0	4.9	2.6	CAP	20	100.2	0.1	0.7
	200	100.7	0.3	0.9		200	99.3	1.4	2.9		200	100.7	0.6	1.0
	800	100.0	4.7	0.6		800	98.9	0.9	4.4		800	103.6	0.5	1.0
NPIP	20	98.4	2.4	3.1	NPIP	20	100.3	2.0	5.9	NPIP	20	99.1	3.9	2.2
	200	99.9	0.5	2.0		200	98.5	0.9	0.5		200	103.6	0.3	0.6
	800	98.4	3.4	1.0		800	99.7	2.5	2.8		800	98.5	0.4	0.9
EMP	20	100.3	1.7	1.9	EMP	20	98.6	4.0	2.4	EMP	20	100.1	1.4	1.7
	200	101.2	0.1	2.4		200	98.6	1.7	1.2		200	98.5	1.2	4.7
	800	100.0	1.1	4.1		800	98.8	1.1	2.0		800	99.0	1.5	4.6
MBZ	20	100.4	2.5	0.7	MBZ	20	99.8	5.2	2.2	MBZ	20	102.0	0.4	1.7
	200	99.8	2.2	0.2		200	102.3	3.6	0.6		200	103.5	2.0	1.9
	800	100.2	2.3	0.7		800	103.5	4.1	0.8		800	98.3	0.1	4.6
DIB	20	99.3	0.2	5.0	DIB	20	103.9	1.1	2.2	DIB	20	100.0	0.2	1.4
	200	99.1	1.1	0.1		200	101.6	1.8	3.1		200	98.9	1.4	0.3
	800	98.2	0.8	0.3		800	98.6	2.8	3.2		800	102.1	0.6	2.1
ETS	20	103.0	1.7	2.6	ETS	20	101.1	2.6	2.0	ETS	20	99.0	2.3	3.4
	200	99.2	1.9	1.1		200	100.9	5.0	1.6		200	99.8	1.3	3.9
	800	99.9	2.3	2.7		800	98.6	2.5	2.5		800	101.2	0.9	1.7
6DTBP	20	102.1	0.7	1.3	6DTBP	20	101.2	1.1	1.1	6DTBP	20	104.6	3.4	0.7
	200	98.4	1.2	1.0		200	98.9	1.1	1.9		200	103.0	0.6	1.4
	800	98.5	1.4	0.1		800	98.8	2.7	0.8		800	98.7	0.1	0.9
BBS	20	100.8	0.1	0.3	BBS	20	100.1	1.1	0.5	BBS	20	99.5	3.2	0.9
	200	103.3	0.8	0.8		200	100.0	0.9	2.5		200	103.3	5.2	4.1
	800	99.2	0.4	0.4		800	99.5	0.1	0.4		800	103.4	2.0	4.1
BHT	20	100.3	1.4	0.3	BHT	20	100.5	0.3	1.5	BHT	20	98.6	4.8	1.9
	200	100.9	0.5	2.3		200	101.3	3.1	0.5		200	99.4	3.8	1.1
	800	98.7	0.1	3.1		800	98.7	2.2	2.4		800	98.9	5.2	2.5
BPA	20	99.1	1.3	1.2	BPA	20	99.7	2.4	1.9	BPA	20	104.7	1.7	1.0
	200	102.3	1.1	0.6		200	98.3	0.9	1.2		200	103.8	3.6	2.8
	800	100.1	0.8	1.8		800	104.4	2.0	2.1		800	102.6	4.0	1.7
BHT-CHO	20	99.4	1.7	1.0	BHT-CHO	20	98.2	0.5	3.0	BHT-CHO	20	99.5	0.3	0.6
	200	103.8	0.1	4.8		200	99.6	0.1	3.4		200	100.1	0.6	0.8
	800	99.3	0.6	4.7		800	101.3	3.5	3.5		800	100.1	0.4	0.5
BHT-COOH	20	98.6	4.6	2.7	BHT-COOH	20	98.9	2.2	0.4	BHT-COOH	20	100.0	0.2	2.1
	200	103.8	1.4	1.0		200	99.2	5.3	0.2		200	100.7	0.9	1.3
	800	102.4	1.7	0.2		800	99.1	2.1	0.8		800	98.6	0.8	2.3
TTB	20	99.4	2.9	0.1	TTB	20	99.9	1.5	1.9	TTB	20	99.7	3.6	1.7
	200	100.4	0.5	2.7		200	100.6	3.7	0.1		200	99.4	1.8	4.5
	800	103.6	3.8	0.5		800	100.8	3.0	1.7		800	99.5	1.5	6.1
TBP	20	102.6	1.1	3.7	TBP	20	99.9	1.8	3.1	TBP	20	99.1	0.5	0.6
	200	100.8	3.4	1.6		200	99.7	6.9	2.9		200	103.6	0.8	1.9
	800	104.4	2.7	1.3		800	99.6	3.9	1.8		800	104.1	2.9	7.2

Protein 1					Protein 2					Protein 3				
Compound	Spiked level (ng mL^{-1})	Recovery (%)	Precision (%)		Compound	Spiked level (ng mL^{-1})	Recovery (%)	Precision (%)		Compound	Spiked level (ng mL^{-1})	Recovery (%)	Precision (%)	
			Intra-day	Inter-day				Intra-day	Inter-day				Intra-day	Inter-day
ERU	20	101.0	2.3	1.7	ERU	20	98.0	1.4	1.3	ERU	20	100.5	0.3	0.6
	200	100.2	1.2	3.7		200	99.9	0.8	2.1		200	100.0	0.6	0.8
	800	101.9	0.8	3.3		800	98.0	1.9	0.9		800	101.0	0.4	0.5
ATBC	20	98.9	1.0	1.0	ATBC	20	99.0	4.9	2.1	ATBC	20	101.6	0.2	2.1
	200	99.7	0.6	2.5		200	99.0	0.8	1.8		200	101.1	0.9	1.3
	800	99.1	4.1	2.7		800	99.4	1.2	1.1		800	101.6	0.8	2.3
BBOT	20	98.4	0.1	0.2	BBOT	20	99.8	1.1	1.3	BBOT	20	99.3	3.6	1.7
	200	98.7	4.6	4.7		200	99.4	1.4	2.0		200	100.6	1.8	4.5
	800	103.8	2.1	0.3		800	101.9	1.2	1.5		800	101.0	1.5	6.1
bDtBPP	20	98.2	1.9	3.5	bDtBPP	20	98.3	2.4	3.1	bDtBPP	20	100.6	0.5	0.6
	200	102.5	3.3	2.1		200	100.9	5.0	2.2		200	99.7	0.8	1.9
	800	104.0	3.6	2.3		800	99.3	1.5	1.5		800	100.8	2.9	7.2

Figure 3S

% Mass recoveries of the spiked elements in both, buffer, and protein materials.

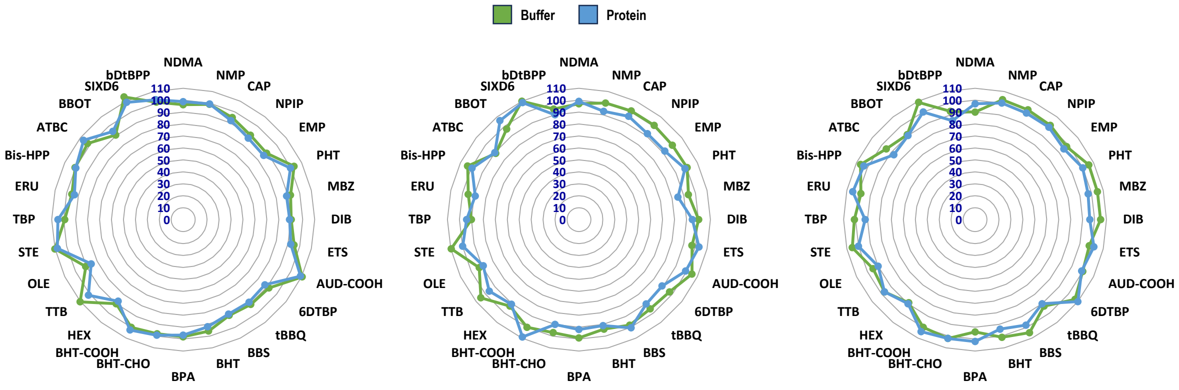

Protein 1 **Protein 2** **Protein 3**

Figure 4S

Structures from organic compounds that are highly polar despite their high MW.

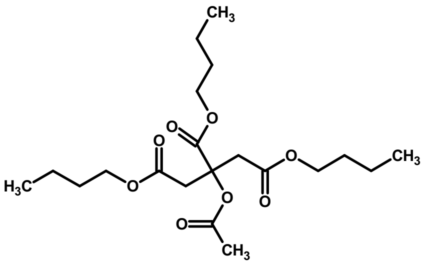

ATBC
MW = 402.48
Log P = 3.53

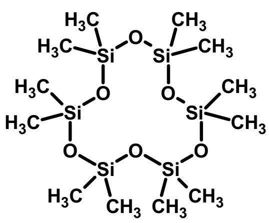

SIXD6
MW = 444.92
Log P = 1.15

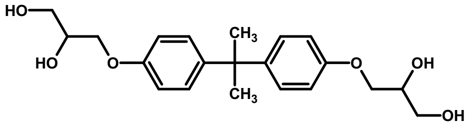

Bis-HPP
MW = 376.44
Log P = 1.70

Table 6S

Experimental sieving coefficients (*)

(a) Training set

Compound	P1	P2	P3
NDMA	> 0.843	> 0.848	> 0.829
NMP	> 0.822	> 0.817	> 0.819
CAP	> 0.779	> 0.709	> 0.707
NPIP	> 0.728	> 0.730	> 0.731
EMP	> 0.693	> 0.699	> 0.696
MBZ	> 0.616	> 0.799	> 0.667
DIB	> 0.609	> 0.531	> 0.551
ETS	> 0.668	> 0.652	> 0.603
6DTBP	0.398	0.403	0.393
BBS	> 0.588	> 0.571	> 0.635
BHT	0.315	0.351	0.365
BPA	0.496	0.521	0.496
BHT-CHO	0.445	0.436	0.441
BHT-COOH	0.470	0.499	0.464
TTB	0.271	0.246	0.221
TBP	0.437	0.409	0.427
ERU	0.192	0.172	0.172
ATBC	0.439	0.435	0.480
BBOT	0.205	0.211	0.211
bDtBPP	0.176	0.154	0.154

(b) Prediction set

Compound	P1	P2	P3
PHT	> 0.963	> 0.969	> 0.970
AUD-COOH	> 0.721	> 0.708	> 0.715
tBBQ	0.434	0.413	0.460
HEX	0.369	0.324	0.313
OLE	0.292	0.281	0.320
STE	0.233	0.264	0.249
Bis-HPPP	> 0.596	> 0.628	> 0.602
SIXD6	> 0.707	> 0.723	> 0.667

(*) Compounds with concentrations below the LOQs were indicated with "> sieving coefficient", and the LOQ concentration of the UF2 retentate was used for calculations.

Table 7S
Model parameters from Figure 6.

(b) PCA Loadings plot

Primary ID	p[1]	p[2]
pH	0.003	0.018
MW	-0.358	0.367
Solubility	0.313	0.314
Log P	-0.387	-0.252
Log D	-0.391	-0.277
Polarizability	-0.391	0.241
SASA	-0.380	0.268
TPSA	-0.126	0.652
S	0.398	0.267

(d) OPLS-DA: VIP

Primary ID	VIP_{pred}
Log D	1.241
Solubility	1.201
Log P	1.154
Polarizability	1.086
MW	1.055
SASA	1.003
TPSA	0.621
pH	0.056

(e) OPLS-DA: Coefficient

Primary ID	CoeffCS[1](Class 1)	CoeffCS[1](Class 2)
pH	0.034	-0.034
MW	0.294	-0.294
Solubility	0.171	-0.171
Log P	-0.707	0.707
Log D	-0.582	0.582
Polarizability	0.340	-0.340
SASA	0.215	-0.215
TPSA	-0.537	0.537

(g) OPLS: VIP

Primary ID	VIP_{pred}
Log D	1.235
Log P	1.231
Polarizability	1.160
Solubility	1.124
MW	1.089
SASA	1.020
TPSA	0.355
pH	0.009

(h) OPLS: Coefficient

Primary ID	CoeffCS[1](S)
pH	0.001
MW	-0.110
Solubility	0.229
Log P	-0.268
Log D	-0.261
Polarizability	-0.159
SASA	-0.090
TPSA	0.164

Table 8S

Clearance percentages from Figure 7.

Compound	MW	Log P	S(*)	Clearance (%)			
				P1	P2	P3	Average
NDMA	74.08	0.04	0.849	99.97	99.98	99.98	99.98
NMP	99.13	-0.36	0.812	99.97	99.98	99.98	99.98
CAP	113.16	0.31	0.798	99.97	99.95	99.98	99.97
NPIP	114.15	0.89	0.731	99.95	99.98	99.99	99.97
EMP	121.18	1.85	0.693	99.95	99.95	99.97	99.96
PHT	166.13	1.29	0.967	99.92	99.99	99.96	99.96
MBZ	167.24	2.88	0.584	99.87	99.98	99.99	99.95
DIB	197.28	3.26	0.609	99.97	99.95	99.99	99.97
ETS	199.27	1.67	0.647	99.97	99.97	99.98	99.97
AUD-COOH	201.31	0.23	0.715	99.94	99.93	99.95	99.94
6DTBP	206.32	4.76	0.398	99.71	99.94	99.94	99.86
BBS	213.30	2.13	0.608	99.98	99.98	99.99	99.98
tBBQ	220.31	3.88	0.436	99.88	99.94	99.90	99.91
BHT	220.35	5.27	0.351	99.76	99.89	99.90	99.85
BPA	228.29	4.05	0.496	99.83	99.94	99.94	99.90
BHT-CHO	234.34	4.47	0.445	99.74	99.89	99.91	99.85
BHT-COOH	250.33	4.42	0.517	98.73	99.95	99.96	99.55
HEX	255.44	5.45	0.335	98.71	97.77	97.92	98.13
TTB	262.44	6.31	0.271	98.96	98.98	98.96	98.97
TBP	266.32	4.09	0.437	99.60	99.88	99.94	99.81
OLE	281.48	6.00	0.298	97.99	98.00	98.57	98.19
STE	284.48	7.15	0.249	95.78	95.89	95.71	95.79
ERU	337.59	7.76	0.192	93.98	93.79	94.27	94.01
Bis-HPPP	376.44	1.70	0.609	99.97	99.98	99.94	99.96
ATBC	402.48	3.53	0.439	99.27	99.67	99.87	99.60
BBOT	430.56	7.58	0.205	94.07	93.23	93.72	93.67
SIXD6	444.92	1.15	0.699	99.30	99.50	99.40	99.40
bDtBPP	474.62	9.23	0.229	93.89	93.53	93.72	93.71

(*) S corresponds to the average value from the 3 proteins, according to Table 6S.

Red font indicates compunds from the prediction set.
